# Quantitative Proteomic Analysis of Wheat Seeds during Artificial Ageing and Priming Using the Isobaric Tandem Mass Tag Labeling

**DOI:** 10.1371/journal.pone.0162851

**Published:** 2016-09-15

**Authors:** Yangyong Lv, Shuaibing Zhang, Jinshui Wang, Yuansen Hu

**Affiliations:** College of Biological Engineering, Henan University of Technology, Zhengzhou, China; Wuhan Botanical Garden, CHINA

## Abstract

Wheat (*Triticum aestivum* L.) is an important crop worldwide. The physiological deterioration of seeds during storage and seed priming is closely associated with germination, and thus contributes to plant growth and subsequent grain yields. In this study, wheat seeds during different stages of artificial ageing (45°C; 50% relative humidity; 98%, 50%, 20%, and 1% Germination rates) and priming (hydro-priming treatment) were subjected to proteomics analysis through a proteomic approach based on the isobaric tandem mass tag labeling. A total of 162 differentially expressed proteins (DEPs) mainly involved in metabolism, energy supply, and defense/stress responses, were identified during artificial ageing and thus validated previous physiological and biochemical studies. These DEPs indicated that the inability to protect against ageing leads to the incremental decomposition of the stored substance, impairment of metabolism and energy supply, and ultimately resulted in seed deterioration. Kyoto Encyclopedia of Genes and Genomes (KEGG) analysis revealed that the up-regulated proteins involved in seed ageing were mainly enriched in ribosome, whereas the down-regulated proteins were mainly accumulated in energy supply (starch and sucrose metabolism) and stress defense (ascorbate and aldarate metabolism). Proteins, including hemoglobin 1, oleosin, agglutinin, and non-specific lipid-transfer proteins, were first identified in aged seeds and might be regarded as new markers of seed deterioration. Of the identified proteins, 531 DEPs were recognized during seed priming compared with unprimed seeds. In contrast to the up-regulated DEPs in seed ageing, several up-regulated DEPs in priming were involved in energy supply (tricarboxylic acid cycle, glycolysis, and fatty acid oxidation), anabolism (amino acids, and fatty acid synthesis), and cell growth/division. KEGG and protein-protein interaction analysis indicated that the up-regulated proteins in seed priming were mainly enriched in amino acid synthesis, stress defense (plant-pathogen interactions, and ascorbate and aldarate metabolism), and energy supply (oxidative phosphorylation and carbon metabolism). Therefore, DEPs associated with seed ageing and priming can be used to characterize seed vigor and optimize germination enhancement treatments. This work reveals new proteomic insights into protein changes that occur during seed deterioration and priming.

## Introduction

Wheat (*Triticum aestivum* L.), one of the most important, oldest and widely cultivated crops, is a staple food source for humans and livestock feed worldwide because of its high nutritional value [1, 2]. As orthodox type seeds, wheat seeds undergo desiccation after maturation, which enables to survive for a long time in a metabolic standstill situation [3]. As storage time is prolonged, seed vigor gradually decreases, and the germination rate eventually diminishes; as a consequence, commercial and genetic losses occur [4, 5]. Hence, seed ageing and germination mechanisms should be understood to develop new measures for seed conservation and production.

Seed ageing causes the physiological deterioration of seeds, which includes a reduced germination rate and an increased post-germination growth time [6, 7]. At present, the altered physiological and biochemical characteristics of seeds have been extensively investigated to elucidate seed aging mechanisms [8, 9]. Seed deterioration is mainly influenced by the accumulation of reactive oxygen species, lipid peroxidation mediated by free radicals, disruption of cellular membranes, and damage to proteins and nucleic acids [7, 8, 10–15]. Proteomic studies on artificially-aged *Arabidopsis* and *Zea mays* (maize) seeds indicated that differentially expressed proteins (DEPs) are mainly involved in oxidative stress, metabolism, and energy supply, which indicated that the proteomic changes can occur during deterioration at the dry state of aged seeds [4, 7]. Das and Sen-Mandi [16] further revealed that the physiological deterioration of wheat begins in its embryo, and this phenomenon is correlated with germination. Nevertheless, the mechanism underlying artificial ageing of wheat seeds remains unknown.

Crop seed germinability is a vital factor that contributes to seedling performance, plant establishment, and subsequent crop development and growth. Seed germination is controlled by both internal and external factors, including genetics, seed structure, seed chemistry, humidity, and temperature [17]. To improve and synchronize seed germination and emergence, researchers apply seed invigoration treatments called seed priming. Seed priming involves pretreatments with water and various chemical reagents, including polyethylene glycol, ascorbic acid, hormones, and vitamins [18, 19, 20, 21]. Proteomic investigations have been conducted during the seed germination of several plant seeds, such as wheat [22, 23], alfalfa [21], *Arabidopsis* [3], and maize [24]. These proteomic studies, conducted using two-dimensional (2-D) electrophoresis [21, 22] and 2-D differential gel electrophoresis [23], have provided critical information on the metabolic process of seed germination. However, 2-D-gel-based approaches suffer from low reproducibility and under-representation of low abundance and hydrophobic proteins [25]. These limitations can be overcome by a non-gel-based quantitative proteomic approach using isobaric tagging reagents. Isobaric tagging reagents, such as tandem mass tags (TMT) and isobaric tags for relative and absolute quantification (iTRAQ), have been developed for mass spectrometry (MS)-based protein detection and quantification in complicated samples [26, 27]. For instance, iTRAQ has been applied to conduct a quantitative proteomics study on wheat grain development and drought response [28, 29]. However, quantitative proteomics studies on wheat seed priming have yet to be reported.

The Chinese wheat cultivar ‘Aikang58’, a medium-hard wheat widely cultivated in the main wheat production areas of China, exhibits excellent characteristics, including drought tolerance, freeze resistance, wide adaptability, and high yield [30]. In this work, the first TMT-based quantitative proteome analysis of elite Chinese wheat cultivar ‘Aikang58’ seeds was conducted during artificial ageing and priming. We uncovered new information on the proteomic changes during ageing and priming in wheat seeds that might provide new insights into metabolic pathways, as well as into adverse defense mechanisms during seed deterioration and priming.

## Materials and Methods

### Wheat seeds, artificial ageing and priming treatment

The elite Chinese bread wheat cultivar ‘Aikang 58’ seeds used in this study were purchased from the Henan Academy of Agricultural Science. Seeds with similar sizes and weights were selected, and the germination rate (Gr) was calculated in accordance with the methods proposed by Dong et al. [23]. The original Gr was 98.0% (designated as WH98), and the seed moisture content was 9.79%.

Seeds were artificially aged by sealing them in air-tight plastic bottles and then stored at 45°C (± 1°C) and 50% relative humidity in a constant-temperature- and humidity-controlled cabinet (Binder KMF720, Tuttingen, Germany) in accordance with previous described methods [4] with minor modification. The vigor of the wheat seeds was determined regularly. The seed samples with three biological replicates were collected when Gr were 50%, 20%, and 1% (designated as WH50, WH20, and WH01 respectively). The collected samples were stored at -80°C prior to analysis. Unaged seeds (WH98) were used as control specimens.

Hydro-priming treatment was based on a previous method [3]. In brief, hydro-primed seeds (designated as WH100) were prepared by immersing dry mature seeds in sterilized water for 8 h at 25°C, packed in a wet gauze, and incubated for 12 h at 25°C.

### Scanning electron microscopy (SEM)

SEM analysis was performed on the basis of a previous method [31]. WH98, WH50, WH20, and WH01 were halved vertically to the ventral side by using a cryostat (Leica CM1950, Solms, Germany). The cut side of embryo of the aged seeds was examined under a SEM (Quanta 250 FEG, FEI, Hillsboro, OR, USA).

### Protein extraction and trypsin digestion

Embryo samples were collected by the dissection of WH98, WH50, WH20, WH01, and WH100 seeds as previously described [32], with each having three biological replicates. Afterward, the embryo samples of the wheat seeds were sonicated thrice on ice using a high intensity ultrasonic processor (Scientz, Ningbo, China) in lysis buffer (8 M urea, 1% Triton-100, 10 mM dithiothreitol and 1% protease inhibitor cocktail VI). After the samples were centrifuged at 20,000 × *g* for 10 min at 4°C, the supernatant was precipitated with 15% cold trichloroacetic acid for 2 h at -20°C and then centrifuged for 10 min at 4°C. The obtained precipitate was washed with cold acetone thrice, and re-dissolved in buffer (8 M urea and 100 mM triethylammonium bicarbonate, pH 8.0). Protein concentration was determined by using a 2-D Quant kit (GE Healthcare) according to the manufacturer’s instructions, and then stored at -80°C for further use.

Approximately 100 μg of proteins for each sample was digested with trypsin for the subsequent experiments. In briefly, proteins from wheat seed embryos were reduced with 10 mM dithiothreitol for 1 h at 37°C and alkylated with 20 mM indole-3-acetic acid for 45 min at room temperature in the dark. Finally, trypsin was added at 1:50 trypsin:protein mass ratio for the first digestion overnight and at a 1:100 trypsin:protein mass ratio for a second 4-h digestion.

### TMT labeling and high-performance liquid chromatography (LC) fractionation

After trypsin digestion was completed, peptides were desalted using a Strata X C18 SPE column (Phenomenex, Torrance, CA, USA) and vacuum-dried. Peptides were reconstituted in 0.5 M triethylammonium bicarbonate and labeled. In brief, one unit of TMT reagent (labeled 100 μg of protein) was thawed and reconstituted in 24 μl acetonitrile. The peptide mixtures were incubated for 2 h at room temperature, pooled, desalted, and dried through vacuum centrifugation. Five samples with three biological replicates were labeled with TMT tags. WH100 and WH98 were labeled with 130 and 129 respectively. WH50, WH20, and WH01 were labeled with 128, 127 and 126respectively.

The TMT labeled samples were then fractionated through high pH reverse-phase high-performance liquid chromatography (LC) by using Agilent 300 Extend C18 columns (5 μm particles, 4.6 mm ID, 250 mm length). The LC gradient was run with 2% to 60% acetonitrile in 10 mM ammonium bicarbonate (pH 10) for 80 min to generate 80 fractions. Afterward, which all of the fractions were combined into 18 fractions. The fractionated samples were dried through vacuum centrifugation and stored at -20°C.

### LC-MS/MS analysis

LC-MS/MS was performed on the basis of a previous report [33]. In brief, the peptides were dissolved in 0.1% formic acid, directly loaded onto a reversed-phase pre-column (Acclaim PepMap 100, Thermo Fisher Scientific, Waltham, MA, USA), and separated using a reversed-phase analytical column (Acclaim PepMap RSLC, Thermo Fisher Scientific). The gradient was increased from 7% to 20% solvent B (0.1% formic acid in 98% acetonitrile) for 24 min, from 20% to 35% in 8 min, and from 35% to 80% in 5 min. The gradient was then maintained at 80% for the last 3 min. A constant flow rate of 280 nl/min was set in an EASY-nLC 1000 UPLC system. The resulting peptides were analyzed by using Q Exactive^™^ hybrid quadrupole-Orbitrap mass spectrometer (Thermo Fisher Scientific).

The peptides were subjected to a nanospray ion source followed by tandem mass spectrometry (MS/MS) in Q Exactive^™^ (Thermo Fisher Scientific) coupled online to ultra performance LC. The peptides were then selected for MS/MS by using the NCE settings of 27, 30, and 33. A data-dependent procedure that alternated between one MS scan followed by 20 MS/MS scans was applied to the top 20 precursor ions above a threshold ion count of 1.0E4 in the MS survey scan with a 30.0 s dynamic exclusion. The fixed first mass was set at 100 *m/z*. The electrospray voltage and *m/z* scan range were described previously [33].

### Database search and bioinformatics analysis

The resulting MS/MS data were processed using Mascot search engine (v.2.3.0 http://www.matrixscience.com/). Tandem mass spectra were used as query in the *Uniprot_Triticum_aestivum* database (100,981 sequences). Trypsin/P was specified as the cleavage enzyme that allows up to two missing cleavage sites. The mass error was set to 10 ppm for precursor ions and 0.02 Da for fragment ions. Carbamidomethyl on cysteine, TMT-6plex (N-term), and TMT-6 plex (K) were specified as fixed modifications, and oxidation on methionine was specified as a variable modification. The false discovery rate was adjusted to < 1%, and the peptide ion score was set at > 20. The proteins displaying a 1.2 fold change between artificially aged and normal seeds (WH01 *vs* WH98, WH20 *vs* WH98 and WH50 *vs* WH98) and between priming and normal seeds (WH100 *vs* WH98) were considered as DEPs if p < 0.05.

### Protein annotation and functional analysis

Gene ontology (GO) (http://www.geneontology.org) and Kyoto Encyclopedia of Genes and Genomes (KEGG) (http://www.genome.jp/kegg/pathway.html) analyses were conducted in accordance with previously reported methods [34]. Proteins were subjected to a eukaryotic orthologous group (KOG) analysis by performing a homology search of the KOG database with the following parameters: E value < 1e-5, identities > 80%, and percent of match length > 60%. A domain annotation was performed using InterProScan on the InterPro (http://www.ebi.ac.uk/interpro/) domain database via Web-based interfaces and services [35]. Subcellular localization was determined by Wolfpsort (version of PSORT/PSORT II, http://psort.hgc.jp/).

GO, KEGG pathway, and protein domain enrichment analyses were performed, and a two-tailed Fisher’s exact test was employed to examine the enrichment of the DEPs against all of the identified proteins. Multiple hypothesis testing was corrected by using standard false discovery rate control methods, and domains with a corrected p-value < 0.05 were considered significant.

For hierarchical clustering based on different protein functional classifications (GO, domain, pathway), proteins from the categories were obtained after enrichment and then the categories were filtered to identify those that were at least enriched in one of the clusters with p-value < 0.05. This filtered p-value matrix was transformed by using the function *x* = −log10 (p-value), and *x* values were z-transformed for each functional category. Cluster membership was visualized by using a heat map via the “heatmap.2” function from “gplots” R-package.

### Protein-protein interaction (PPI) network

All of the DEPs were used as query against the STRING database (version 10.0, http://string-db.org) [36] to identify protein-protein interactions. STRING defines a metric called “confidence score” to describe the interaction confidence. We obtained all of the interactions with a confidence score ≥ 0.7 (high confidence). The interaction network from STRING was visualized with Cytoscape (http://www.cytoscape.org/) [37]. Molecular complex detection (MCODE) was utilized to analyze densely connected regions. MCODE is part of the plug-in toolkit of the network analysis and visualization software Cytoscape.

### RNA isolation and quantitative real-time PCR (qRT-PCR) analysis

Total RNA from wheat embryos of WH98, WH50, WH20, and WH01 were extracted by using RNAiso Plus reagent (Takara, Tokyo, Japan), and genomic DNA was removed by treating with DNase I (Takara) following the manufacturer’s protocol. Reverse transcription was performed by using a PrimeScriptRT reagent kit (Takara, RR047A) with a random primer mix in accordance with the manufacturer’s instructions. Primer pairs for qRT-PCR analysis (S3 Table) were designed by Primer5 and checked by querying the primer sequences, using the BLAST algorithm against the NCBI database. All of the primers were consistent with their respective target gene sequences. qRT-PCR of the translationally-controlled tumor protein, (TCTP; Q8LRM8), asparagine synthetase (AS; W5CK94) and catalase (CAT; W5HND1) genes was performed using Mastercycler ep realplex (Eppendorf, Hamburg, Germany) and SYBR Premix Ex Taq (Takara, RR420A) with ADP-ribosylation factor as a reference gene [38].

## Results

### Germinability and microstructural changes during artificial ageing of wheat seeds embryo

**Fig 1:**
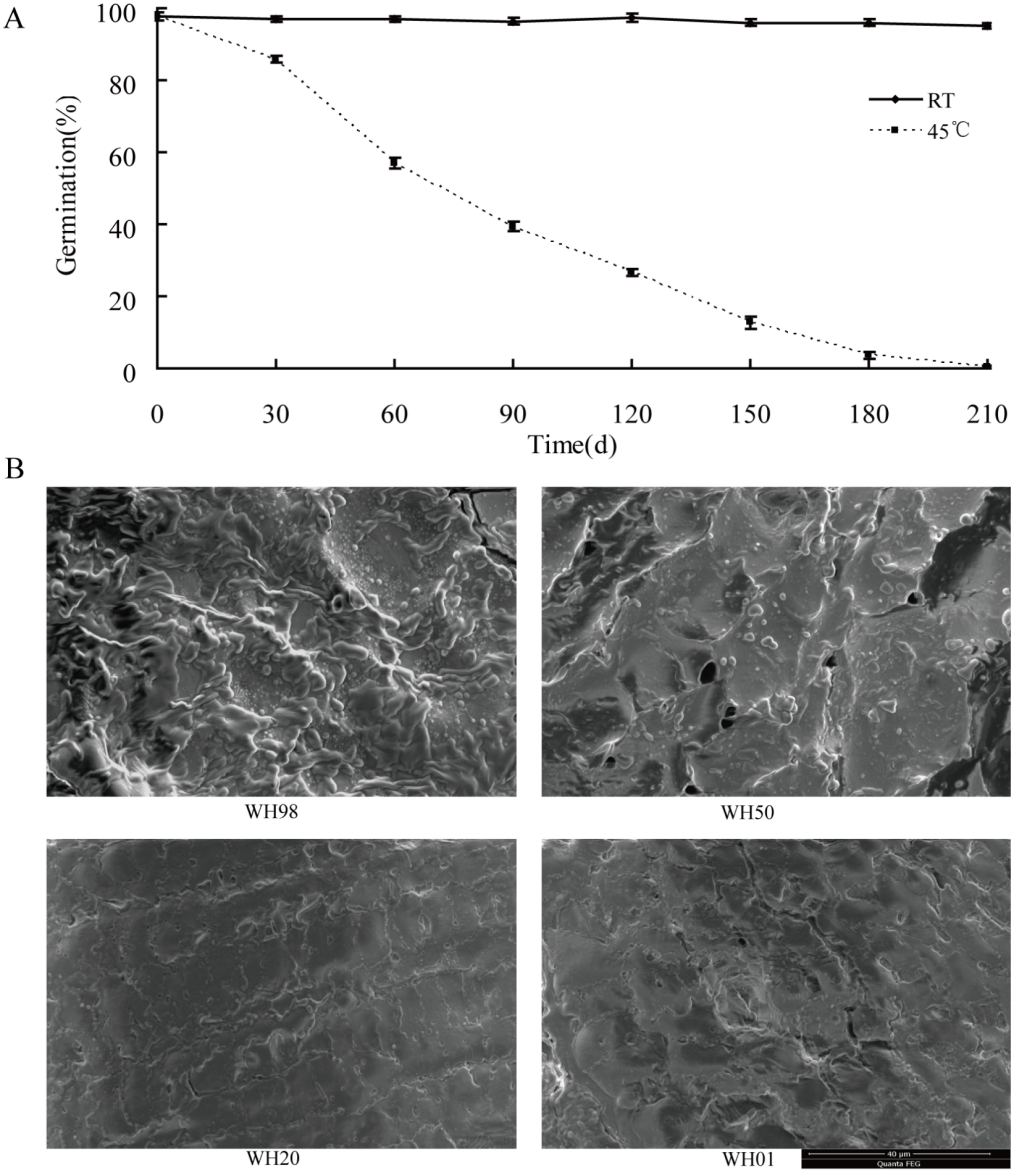
Wheat seed development during the artificial ageing of cultivar ‘Aikang58’. (A) Seed germination during artificial ageing. (B) Scanning electronic microscope observations of embryos from artificial ageing grains.

To investigate seed vigor and structural changes in embryos, Gr and SEM were employed to evaluate the alterations. There was no obvious difference in the Gr of seeds stored at room temperature for nearly 210 days, while the artificial ageing treatment resulted in a decreased germinability from 98% to 50% (65 d), 20% (131 d), and 1% (210 d). Gr was decreased rapidly from 30 (85%) to 90 d (41%), and almost no seeds could germinate after being stored 210 d (1%) at 45°C (Fig 1A). Gr also indicated that the deterioration rate accelerated after 30 d at 45°C. After priming was performed, the radicle protruded through the seed coat and the embryo was collected for further analysis (S1A Fig).

The SEM images (magnification was 4000×) of the cut side of embryos during artificial ageing were shown in Fig 1B. The results indicated that artificially aged seeds (WH50, WH20, and WH01) showed a degradation of granules and fold change on the surface compared with those of WH98. The altered microstructure and decreased Gr suggested that various physiological reactions might occur during this phase.

### Overview of quantitative proteomics analysis

In this study, the TMT-based quantitative proteomic characteristics of the ‘Aikang58’ cultivar were investigated to uncover the altered proteins involved in artificial deterioration and priming. A global profile of the quantitative proteome was obtained by using the embryos from WH98, WH50, WH20, WH01 and WH100, with three biological replicates each. The SDS-PAGE analysis of proteins from the embryo was shown in S1B Fig. Each lane was loaded with 10 μg of proteins. When samples were subjected to an LC-MS/MS analysis, the data validation was evaluated. The distribution of mass error is near zero and most errors are less than 0.02 Da. These findings indicated that the MS data’s accuracy met the requirement (S1C Fig). The lengths of most of the peptides were distributed between 8 and 16 aa, which was consistent with the properties of tryptic peptides (S1D Fig), indicating that the sample preparation met the standard.

A total of 6281 proteins were identified, of which 3574 proteins with quantitative information were elicited from *T*. *aestivum* in the three biological replicates (S1 Table), and the coverage of identified proteins among the three biological repeats are also presented (S2A Fig). The protein expression levels were comparatively analyzed and divided into two groups: protein changes occurring during artificial ageing (WH01 *vs* WH98, WH20 *vs* WH98, and WH50 *vs* WH98), and protein changes occurring during priming (WH100 *vs* WH98). Pearson’s correlation coefficient was obtained to evaluate the repeatability of protein relative quantitation. The pair-wise Pearson’s correlation coefficients of the samples (protein changes during artificial ageing) are presented in a red-white-green heat map format (S2B Fig) and the reproducibility of the protein quantitation (protein changes during priming) of the samples is presented (S2C Fig). A 45°-diagonal line was obtained throughout the detection range, and this finding indicated the expected distribution without obvious changes among the three biological replicates (artificial ageing). The correlation coefficient was > 0.82 among the three biological replicates (priming), which indicated good reproducibility.

**Fig 2:**
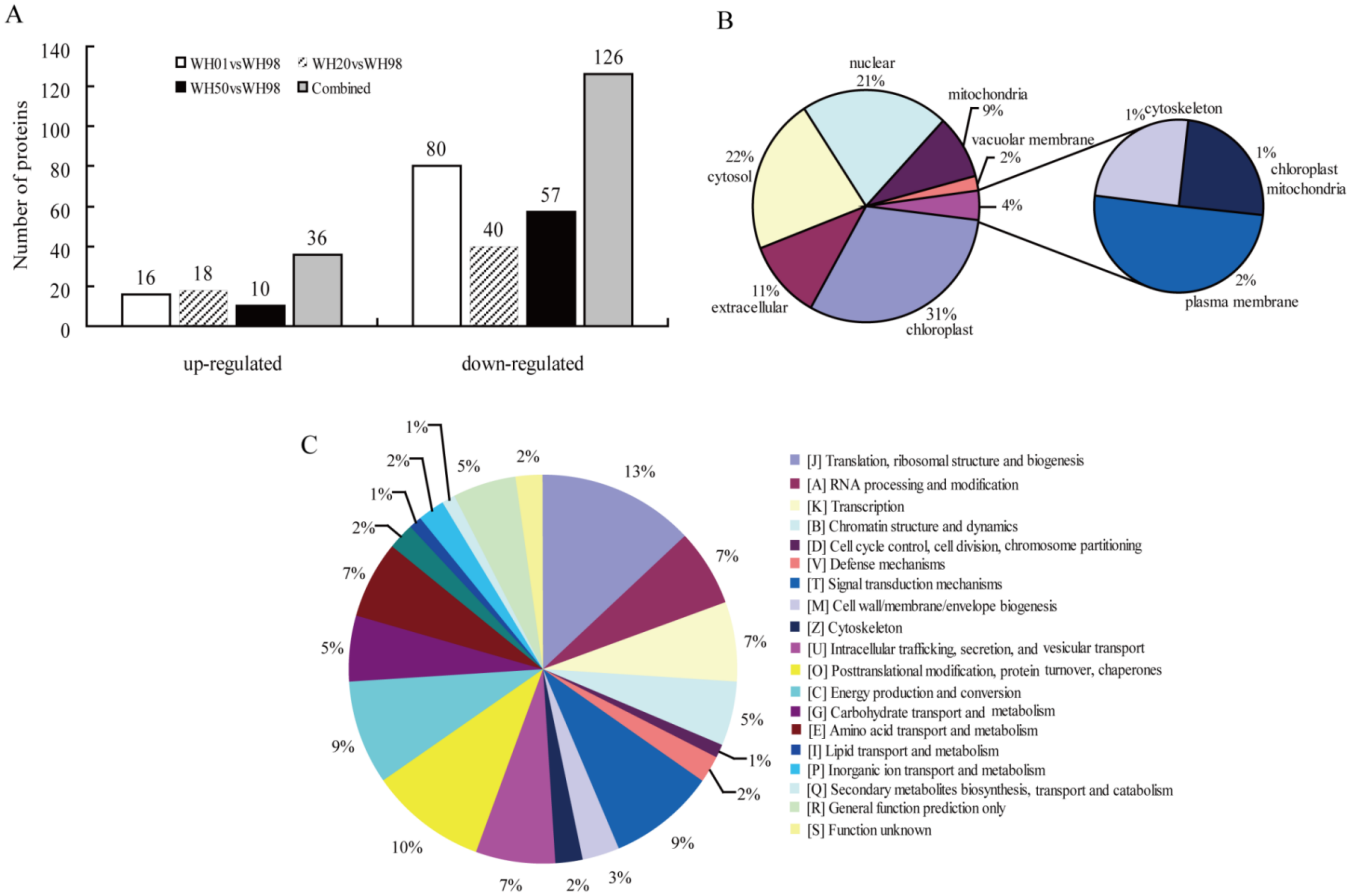
Differentially expressed proteins and their functional classification analysis during artificial ageing. (A) Differentially expressed proteins during artificial ageing compared with unaged seeds; (B) sub-cellular localization analysis; (C) eukaryotic orthologous group (KOG) analysis.

All the identified proteins were annotated (S2 Table), including GO, KEGG, KOG, and domain annotations. Of the 3574 quantified proteins, 162 were identified as DEPs, containing 36 up-regulated (≥1.2-fold, p-value ≤ 0.05) and 126 down-regulated (≤ 0.83-fold, p-value ≤ 0.05) in at least one of the artificial ageing stages compared with unaged seeds (Fig 2A, S3 Table). The 36 up-regulated proteins included 16 DEPs at WH01, 18 DEPs at WH20, and 10 DEPs at WH50, of which 8 DEPs were at two stages, meanwhile the 126 down-regulated proteins included 80 DEPs at WH01, 40 DEPs at WH20, and 57 at WH50, of which 10 DEPs were shared by all of the artificial ageing stages and 32 DEPs were shared by two stages (12 at WH01 and WH20, 12 at WH01 and WH50, and 8 at WH20 and WH50).

Subcellular localization revealed the following: 31% of DEPs in the chloroplast, 22% of DEPs in the cytosol, 21% of DEPs in the nucleus, 11% of DEPs in the extracellular, and 9% of DEPs in the mitochondria (Fig 2B). The KOG annotation showed that DEPs were mainly involved in translation, ribosomal structure and biogenesis (13%), post-translational modification, protein turnover, chaperones (10%), energy production and conversion (9%), and signal transduction mechanisms (9%) (Fig 2C). GO analysis demonstrated that DEPs were mainly involved in binding, metabolic process, catalytic activity, and cellular process (S3A Fig).

To further understand the DEPs during artificial ageing, a functional enrichment analysis was performed (S4 Table). A Fisher’s exact test p-value was obtained to evaluate the enrichment analysis with those of the quantified proteins as references. Our results indicated that the proteins mainly involved in the extracellular region, serine-type endopeptidase inhibitor activity, negative regulation of peptidase, and endopeptidase and peptidase activities were down-regulated (S4A Fig), while the DNA-binding complex, nucleosome, DNA-packaging complex, protein heterodimerization and asparagine metabolism were accumulated in the GO enrichment analysis (S4B Fig). The KEGG pathway enrichment analysis showed that proteins involved in the ribosome were up-regulated, whereas proteins in starch, sucrose, ascorbate and aldarate metabolism were down-regulated (S4C, S5 and S6 Figs). Domain enrichment analysis indicated that the proteins involved in the histone core, histone fold, and asparagines synthase were over-represented in the up-regulated proteins, while proteins in the bifunctional inhibitor/plant lipid transfer protein/seed storage helical domain and bifunctional trypsin/alpha-amylase inhibitor helical domain were significantly over-represented in the down-regulated proteins (S4D and S4E Fig).

**Fig 3:**
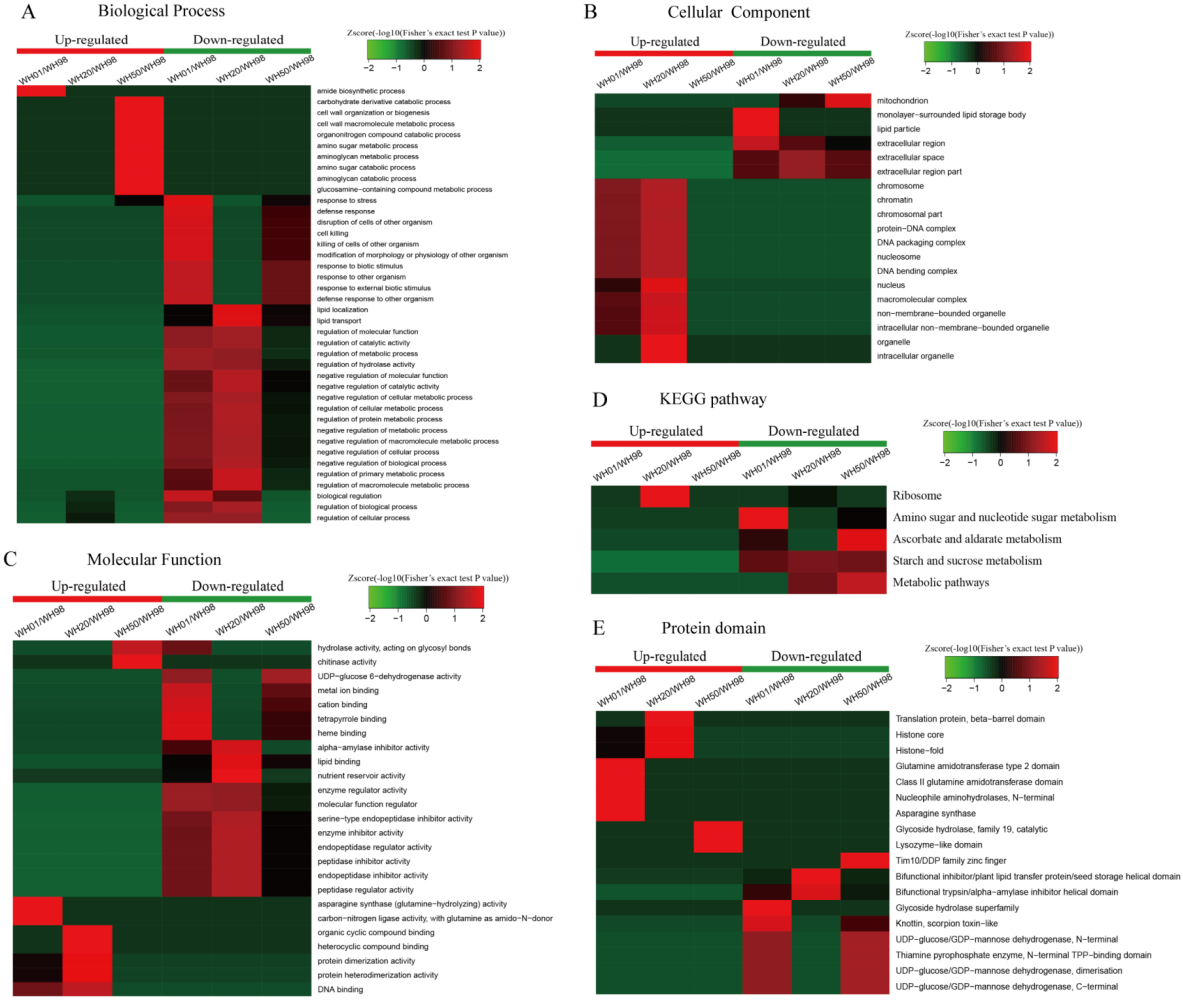
Functional enrichment-based clustering of protein groups during artificial ageing. (A) Biological process; (B) Cellular component; (C) Molecular function; (D) KEGG pathway; (D) Protein domain.

To better understand the dynamics of DEPs during artificial ageing, a hierarchical clustering analysis was performed to obtain the dynamic expression patterns. The up-regulated proteins, including carbohydrate derivatives, organonitrogen compounds, amino sugars, and aminoglycans, in the biological process category (Fig 3A) were mainly enriched in energy catabolic processes at the beginning of artificial ageing (from WH98 to WH50). With an extended artificial ageing time (WH01), these proteins were mainly enriched in amide biosynthesis. The down-regulated proteins were mainly implicated in defense- and stress-related pathways, and over-represented when Gr was nearly zero (WH01). The proteins related to lipid metabolic processes were accumulated when Gr was 20% (WH20). The results also showed that proteins involved in the negative regulation of metabolic processes were enriched in the later stages of artificial ageing (WH20 and WH01). The up-regulated DEPs in the cellular component category (Fig 3B) were mostly implicated in chromatin and nucleus, and were enriched at WH20 and WH01. By contrast, the proteins related to mitochondria and lipids were significantly down-regulated when Gr were 50% (WH50) and 1% (WH01), respectively. The up-regulated DEPs in the molecular function category (Fig 3C) were mainly related to chitinase and hydrolase activities, and clustered at WH50. By comparison, asparagine synthase was accumulated at WH01 and protein dimerization/heterodimerization activity was concentrated at WH20. The down-regulated proteins related to alpha-amylase inhibitor, lipid binding, and nutrient reservoir and enzyme inhibitor activities were enriched at WH20, while endopeptidase and its regulatory activity accumulated at WH20 and WH01. For DEPs in the KEGG pathway (Fig 3D), the up-regulated proteins related to the ribosome accumulated significantly at WH20, whereas the down-regulated proteins related to starch and sucrose metabolism were observed during the whole artificial ageing process. The proteins associated with ascorbate and aldarate metabolism were significantly enriched at WH50, and the amino sugar and nucleotide sugar metabolism were enriched at WH01. For DEPs in the protein domain cluster analysis (Fig 3E), the up-regulated proteins related to translation and histone were clustered at WH20, glutamine amidotransferase and asparagine synthase at WH01, and glycoside hydrolase at WH50. The down-regulated proteins concerned with plant lipid transfer and alpha-amylase inhibitor were enriched at WH20, whereas UDP-glucose/GDP-mannose dehydrogenase accumulated at WH50 and WH01 (Fig 3E).

To illuminate the mechanisms of DEPs that mediated artificial ageing, the PPI network for DEPs were constructed with STRING and Cytoscape. The PPI networks of up and down-regulated proteins were also drawn separately (S7 Fig and S5 Table).

**Fig 4:**
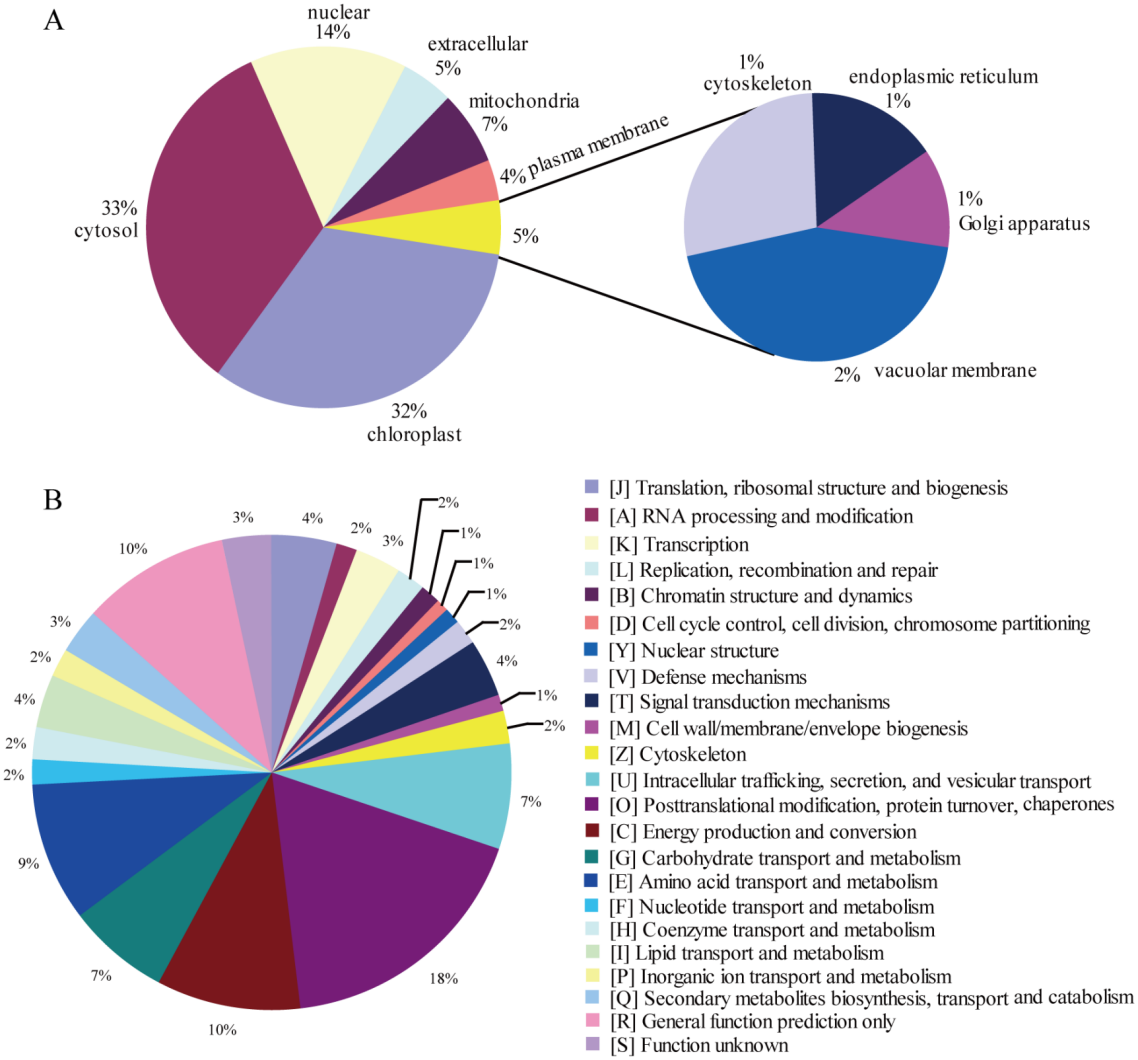
Functional analysis of DEPs during priming. (A) Sub-cellular localization; (B) KOG categorization.

During seed priming, 531 DEPs, including 312 and 219 up-regulated and down-regulated proteins respectively, were presented (S3 Table). Subcellular localization revealed the following: 33% of DEPs were located in the cytosol, 32% of DEPs in the chloroplast, 14% of DEPs in the nucleus, 7% of DEPs in the mitochondria, and 5% of DEPs in the extracellular (Fig 4A). DEPs were mainly classified into five main categories, namely, posttranslational modification, protein turnover, and chaperones (18%); energy production and conversion (10%); amino acid transport and metabolism (9%); carbohydrate transport and metabolism (7%); and intracellular trafficking, secretion, and vesicular transport (7%) (Fig 4B). GO analysis revealed that DEP categories were mainly involved in metabolic process, binding, catalytic activity and cellular process (S3B Fig).

**Fig 5:**
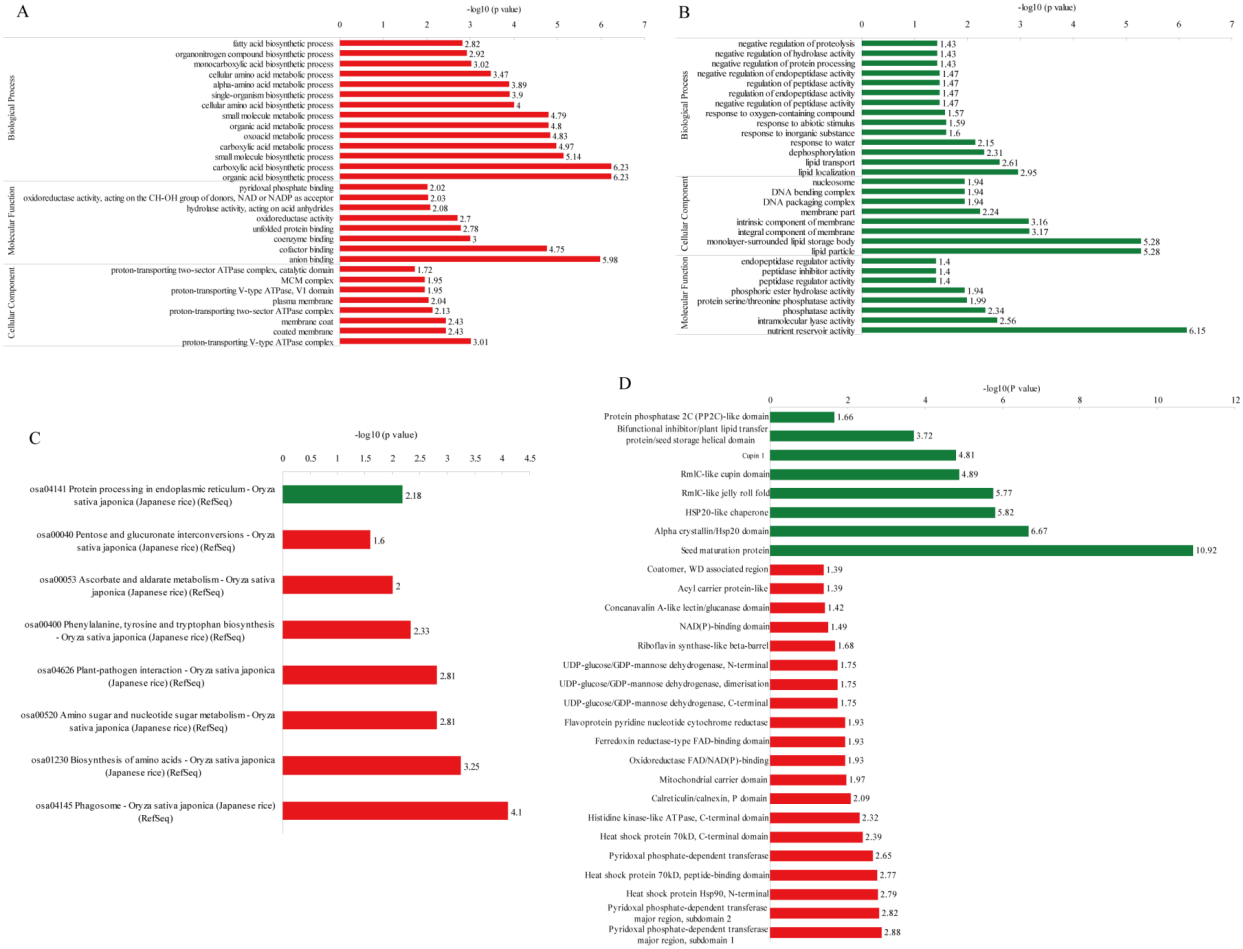
Functional enrichment analysis of DEPs during seed priming. The red bar represents up-regulated proteins, and the green bar represents down-regulated proteins. (A, B) GO enrichment analysis; (C) KEGG enrichment analysis; (D) Protein domain enrichment analysis.

To better investigate the DEPs during seed priming, functional enrichment (GO, KEGG pathway, and protein domain) analyses were performed. A Fisher’s exact test p-value (-log10 [p-value]) was performed to evaluate the enrichment analysis, and the larger the value, the more DEPs were enriched in this category (Fig 5).

The up-regulated proteins significantly accumulated in the anion binding and biosynthetic process categories, including organic acid, carboxylic acid, small molecule, and metabolic processes involving oxoacid and carboxylic acid (Fig 5A). The proteins enriched in each category are listed in detail in S6 Table. In Fig 5B, the down-regulated proteins accumulated in the categories of nutrient reservoir activity, lipid particle and monolayer-surrounded lipid storage body. In Fig 5C, the down-regulated proteins mainly clustered in the protein processing sites in the endoplasmic reticulum (KEGG pathway: S8 Fig) in accordance with the KEGG pathway enrichment analysis. Meanwhile, the enriched KEGG pathways for up-regulated proteins included the phagosome (KEGG pathway: S9 Fig), biosynthesis of amino acids, amino sugar and nucleotide sugar metabolism, plant-pathogen interaction (KEGG pathway: S10 Fig), phenylalanine, tyrosine and tryptophan biosynthesis (KEGG pathway: S11 Fig), ascorbate and aldarate metabolism (KEGG pathway: S12 Fig), and pentose and glucuronate interconversions (KEGG pathway: S13 Fig). The proteins clustered in each pathway are listed in S6 Table. The domain enrichment analysis demonstrated that the down-regulated proteins contained seed maturation protein domains, alpha crystallin/Hsp20 domains, and HSP20-like chaperones. By contrast, the up-regulated proteins contained mainly the pyridoxal phosphate-dependent transferase, 90 kD heat shock protein, and 70 kD heat shock protein peptide-binding domains. The proteins clustered in each domain are listed in S6 Table.

**Fig 6:**
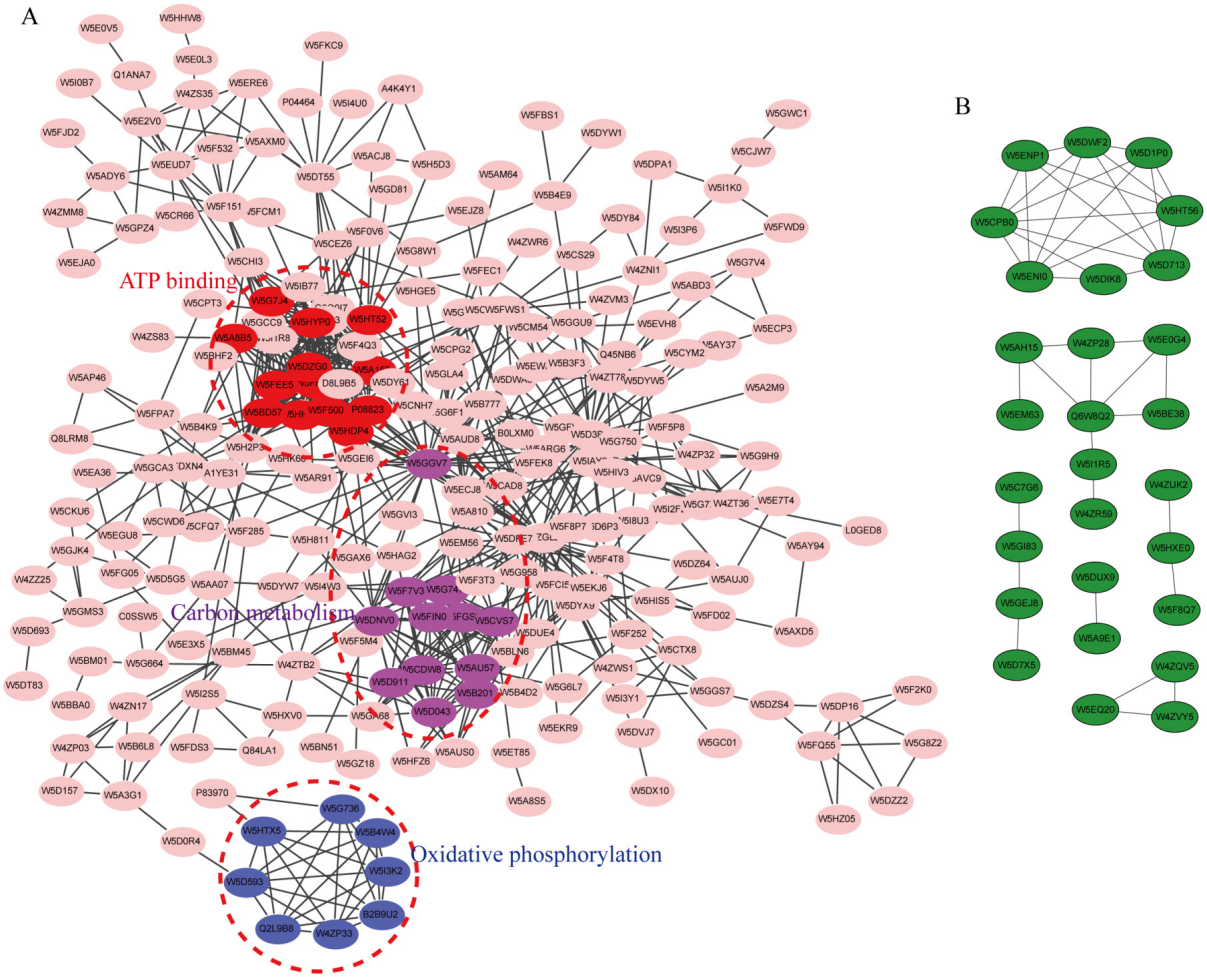
The protein-protein interaction network analysis of up-regulated proteins (A) and down-regulated proteins (B) identified by TMT-labeling during seed priming.

To better elucidate the protein mechanisms involved in seed priming, we constructed the PPI network of DEPs using STRING and Cytoscape. With the MCODE plug-in toolkit, three enriched interaction clusters were associated with oxidative phosphorylation, carbon metabolism, and ATP binding (Fig 6 and S7 Table).

### Differential protein expression during seed artificial ageing

On the basis of protein functions, these DEPs (S3 Table) could be grouped into four categories according to Xin et al. [4], except for the uncharacterized proteins. The four categories included protein destination and storage, metabolism/energy supply, defense/stress, and unclear classification (Table 1).

**Table 1:** Proteins, with annotation information, differentially expressed during seed ageing compared with unaged seeds (WH98) using LC-MS/MS.

Protein accession	Protein description	Protein abundance change	Stage	MW [kDa]	pI
**Protein destination and storage**					
P10385	Glutenin, low molecular weight subunit	0.76,0.78,0.74	WH50,WH20,WH01	42.1	9.04
Q2A784	Avenin-like a1	0.80	WH20	19.7	8.42
P08453	Gamma-gliadin	0.83	WH20	38.4	7.62
Q43659	15kDa grain softness protein	0.83	WH20	21.5	8.02
D2KFG9	Gliadin/avenin-like seed protein	0.81	WH20	24.2	6.2
W5A8E0	60S ribosomal protein L18	1.23	WH20	26.1	11.49
**Metabolism/Energy supply**					
W5A810	Malic enzyme	0.81	WH01	70.9	5.56
W5BLN6	glyceraldehyde-3-phosphate dehydrogenase	0.81	WH50	61.73	6.39
W5CP16	6-phosphofructokinase 1	0.75	WH50	60.60	6.73
W4ZRX8	6-phosphofructokinase 1	0.81	WH50	70.28	6.61
W5B5R3	Sucrose synthase	0.80,0.80	WH20, WH01	103.8	6.06
W5I774	Sucrose synthase	0.81	WH50	103.7	5.72
O04074	Starch branching enzyme1	0.81	WH01	106.3	6.27
**Defense/Stress**					
W5HND1	Catalase	0.82	WH01	59.3	6.57
W5DYW7	L-ascorbate peroxidase	0.82	WH50	31.93	5.85
W5FQ55	UDP-glucose 6-dehydrogenase	0.72,0.67,0.66	WH50,WH20,WH01	62.5	5.84
W5FDW8	UDP-glucose 6-dehydrogenase	0.79,0.75	WH50, WH01	62.5	5.84
P16159	Alpha-amylase/trypsin inhibitor CM16	0.82,0.81	WH20, WH01	17.3	5.31
P16851	Alpha-amylase/trypsin inhibitor CM2	0.82,0.81	WH20, WH01	17.2	6.86
P17314	Alpha-amylase/trypsin inhibitor CM3	0.75,0.75	WH20, WH01	20.0	7.44
P01083	Alpha-amylase inhibitor	0.66,0.78	WH50,WH20	19.2	7.45
M5AJV9	Hemoglobin 1	0.50,0.37	WH50, WH01	21.5	7.85
W5AW62	Oleosin	0.68	WH01	19.9	9.69
W5FHA6	Oleosin	0.81	WH01	18.3	7.06
W5FYW1	Oleosin	0.82	WH01	18.3	7.1
P10969	Agglutinin isolectin 3	0.79	WH01	22.6	6.75
P26913	Probable non-specific lipid-transfer protein	0.76	WH20	4.3	8.54
Q2PCC3	Type 2 non specific lipid transfer protein	0.75	WH50	11.9	9.04
W5FSX7	Non-specific lipid-transfer protein	0.82	WH50	19.4	9.73
P82901	Non-specific lipid-transfer protein 2P	0.67,0.75,0.81	WH50,WH20, WH01	8.2	8.21
W5D2W6	Non-specific lipid-transfer protein	0.81,0.80	WH20, WH01	10.7	9.35
Q8LRM8	Translationally-controlled tumor protein homolog (TCTP)	1.29,1.30	WH20, WH01	22.5	4.55
W5CK94	Asparagine synthetase	1.21	WH01	67.9	6.09
W5FDU3	Asparagine synthetase	1.30	WH01	74.3	6.01
A1YE31	Ribosomal protein L3-A3	1.25	WH20	57.5	9.97
A1YE34	Ribosomal protein L3-B2	1.29	WH20	57.1	10.07
C1K737	Multiprotein bridging factor 1	0.80	WH01	20.3	9.87
**Unclear classification**					
W4ZP38	60S ribosomal protein L36	0.82	WH01	17.7	11.12
W4ZVF9	50S ribosomal protein L16, chloroplastic	0.66	WH50	38.1	10.13
P30569	EC protein I/II	0.56	WH20	9.1	7.85
W5F4N9	DNA-directed RNA polymerase subunit	0.78	WH50	14.7	6.19
W5BFB4	Histone H2B	1.35,1.24	WH20, WH01	23.8	10
W5G4D7	Histone H2B	1.39,1.34	WH20, WH01	24.3	10.05
W5EA78	Histone H2B	1.26	WH20	23.5	10.02
W4ZQV5	Histone H4	1.20	WH20	23.4	11.61

In Table 1, five storage proteins related with wheat flour quality (P10385, Q2A784, P08453, Q43659, and D2KFG9) and one regulatory protein (W5A8E0), were involved in protein destination and storage. The results indicated that storage proteins, except glutenin (P10385), were significantly changed at WH20 in all of the artificial ageing stages (WH50, WH20, and WH01) compared with WH98. Three kinds of proteins related to metabolism/energy, including tricarboxylic acid (TCA) cycle (malic enzyme, W5A810), sucrose synthase (W5B5R3 and W5I774) and starch branching enzyme 1 (O04074) were present and distributed in all of the stages. A total of 24 DEPs that significantly accumulated at the last artificial ageing stages WH20 and WH01 were categorized into defense/stress. These DEPs could be divided into three groups, namely, storage material degradation inhibitors (four alpha-amylase inhibitor and three oleosin proteins); response to oxidative, osmotic, and temperature pressures (catalase, UDP-glucose 6-dehydrogenase, TCTP, and multiprotein bridging factor), and response to biotic stresses (hemoglobin, agglutinin isolectin, five non-specific lipid-transfer proteins, ribosomal protein L3, and two asparagine synthetases). The results also indicated that a considerable number of DEPs involved in defense/stress were down-regulated.

### Differential protein expression during seed priming

According to Dong et al. [23] and Ma et al. [28], DEPs (S3 Table) during seed priming, except the uncharacterized proteins, could be grouped into five categories, namely, energy supply and metabolism, signal transduction/cell growth, protein destination and storage, defense/stress, and unclear classification (Table 2).

**Table 2:** Proteins, with annotation information, differentially expressed during seed priming identified using LC-MS/MS.

Protein accession	Protein description	Protein abundance change	MW [kDa]	pI
**Energy supply**				
W5AU57	Glucose-6-phosphate-dehydrogenase	1.29	66.1	6.02
W5B201	Glucose-6-phosphate-dehydrogenase	1.56	68.8	6.67
W5HFZ6	6-phosphogluconate dehydrogenase	1.43	59.9	5.61
W5FIN0	Triosephosphate isomerase	1.38	37.2	7.04
W5CVS7	Pyruvate kinase	1.41	73.2	9.73
W5A810	Malic enzyme	1.71	70.9	5.56
Q84LA1	Fructan 1-exohydrolase w2	5.21	72.9	4.9
**Metabolism**				
W5DVJ7	Acyl-coenzyme A oxidase	1.45	83.6	6.73
B2ZGL2	Plastid acetyl-CoA carboxylase	1.23	283.3	5.78
W5AXD5	Acyl-[acyl-carrier-protein] desaturase	1.33	50.1	6.04
W5DZ64	Acyl carrier protein	1.25	17.6	5.62
W5F3T3	Acyl carrier protein	2.24	18.7	5.47
W5FD02	Acyl carrier protein	1.24	12.6	4.56
W5H9X1	Reticulon-like proteinER	0.77	28.3	8.16
D8L9B5	Putative PDI-like protein	1.63	56.8	5.36
W5GCC9	Bip	1.46	87.5	5.06
F4Y593	Heat shock protein 90	1.20	99.0	4.98
Q0Q0I7	Heat shock protein 90	1.37	99.5	4.96
W5F285	Eukaryotic translation initiation factor 3 subunit B	1.20	89.8	5.58
W5GCA3	Eukaryotic translation initiation factor 3 subunit F	1.24	35.1	5.09
W5AVC9	Histidinol dehydrogenase, chloroplastic	1.21	57.7	5.73
W5CM54	Cysteine synthase	1.28	38.4	5.51
W5DYX9	Chorismate synthase	1.89	53.8	6.33
W5I8U3	3-phosphoshikimate 1-carboxyvinyltransferase	1.32	47.6	5.37
Q45NB6	Glutamine synthetase (GS1)	2.23	44.7	5.41
W5ARG6	Glutamate dehydrogenase	1.20	50.6	6.2
W5ECP3	N-acetyl-gamma-glutamyl-phosphate reductase	1.57	70.6	9.73
W5DWA6	Adenylosuccinate synthetase	1.56	47.7	5.68
W5F9D7	Lipoxygenase	1.96	106.9	6.1
Q9AXK5	Sucrose-6F-phosphate phosphohydrolase SPP2	0.78	55.9	6.04
B0LXM0	S-adenosylmethionine synthase	1.23	49.6	5.55
W5G6F1	S-adenosylmethionine synthase	4.47	53.8	5.4
**Signal transduction/ Cell growth**				
L0GED8	14-3-3 protein	1.21	33.7	4.83
P04464	Calmodulin	1.32	19.4	4.1
A4K4Y1	Alpha tubulin-2A	1.84	55.0	4.89
W5B4E9	Ubiquitin carboxyl-terminal hydrolase	1.28	94.2	9.14
W5CWD6	Ubiquitin carboxyl-terminal hydrolase	1.22	58.7	5.7
W5D043	Fructose-bisphosphate aldolase	1.59	46.2	8.6
P08823	RuBisCO large subunit-binding protein subunit alpha, chloroplastic	1.46	66.6	4.83
W5B777	Serine hydroxymethyl transferase	1.33	46.0	8.78
W5ECJ8	Serine hydroxymethyltransferase	1.50	68.3	8.24
P83970	Plasma membrane ATPase	1.43	119.1	6.83
W5ETC7	Ferritin	0.82	32.3	5.56
**Protein destination and storage**				
I6QQ39	Globulin-3A	0.47	70.3	8.48
P04730	Gamma-gliadin	0.81	28.4	9.2
P10385	Glutenin, low molecular weight subunit	0.82	42.1	9.04
**Defense/Stress**				
P16347	Endogenous alpha-amylase/subtilisin inhibitor	0.71	21.5	6.77
P01083	Alpha-amylase inhibitor0.28	0.81	19.2	7.45
P16159	Alpha-amylase/trypsin inhibitor CM16	1.22	17.3	5.31
P17314	Alpha-amylase/trypsin inhibitor CM3	1.47	20.0	7.44
P04568	Em protein	0.29	11.6	5.55
P22701	Em protein CS41	0.35	12.0	5.28
P42755	Em protein H5	0.41	11.7	5.14
Q9ZR70	Em protein	0.18	11.6	5.57
W5GEI6	Catalase	1.37	63.1	6.58
W5DP16	UDP-glucose 6-dehydrogenase	1.49	62.6	5.69
W5FDW8	UDP-glucose 6-dehydrogenase	2.23	62.5	5.84
W5FQ55	UDP-glucose 6-dehydrogenase	2.38	62.5	5.84
Q6W8Q2	1-Cys peroxiredoxin	0.76	29.0	6.08
A1YE31	Ribosomal protein L3-A3	1.22	57.5	9.97
Q58A30	P450 (CYP71C6v3)	6.08	65.8	6.77
W5FEK8	Methylenetetrahydrofolate reductase	2.26	76.7	5.46
Q1XHC6	Multidomain cystatin	0.70	31.2	6.37
O64393	Wheatwin-2 (PR4B)	0.81	17.4	8.18
Q8GZB0	Non-specific lipid-transfer protein	0.52	13.6	8.72
P82901	Non-specific lipid-transfer protein 2P	0.67	8.2	8.21
W5FSX7	Non-specific lipid-transfer protein	0.78	19.4	9.73
C1K737	Multiprotein bridging factor 1(MBF1)	0.78	20.3	9.87
Q8LRM8	Translationally-controlled tumor protein homolog (TCTP)	1.68	22.5	4.55
W5FJN1	Chalcone flavanone isomerase	0.76	26.9	5.23
W5GDM5	3-ketoacyl-CoA synthase	1.41	72.4	9.52
W5AW62	Oleosin	0.39	20.0	9.69
W5BE38	Oleosin	0.54	18.2	9.21
W5E9F6	Oleosin	0.66	20.2	9.69
W5FHA6	Oleosin	0.57	18.3	7.06
W5FYW1	Oleosin	0.55	18.3	7.1
**Unclear classification**				
W5IAY2	Ribonucleoside-diphosphate reductase	4.12	92.9	6.2
W4ZVY5	Histone H2A	0.83	14.0	9.55
W5G8W1	Clustered mitochondria protein homolog	1.51	169.3	5.9
W5B3F3	Cytosine-specific methyltransferase	4.42	202.6	5.86
W5CBE3	Glycylpeptide N-tetradecanoyl transferase	1.21	22.7	5.05
W4ZMM8	Coatomer subunit gamma	1.37	111.1	5.03
W5HT52	T-complex protein 1 subunit alpha	1.22	69.5	5.76
W5GAX6	Deoxyhypusine hydroxylase	1.33	36.7	4.78
C0SSW5	Ent-copalyl diphosphate synthase	1.45	98.8	5.88

In Table 2, proteins involved in energy supply could be mainly divided into four functional groups, the pentose phosphate pathway (PPP; W5AU57, W5B201, and W5HFZ6), glycolysis (W5FIN0 and W5CVS7), fatty acid oxidase (W5DVJ7), and TCA cycle (W5A810). The DEPs in metabolism could be mainly divided into three groups, fatty acid synthesis (B2ZGL2, W5AXD5, W5DZ64, W5F3T3, and W5FD02), protein synthesis (W5H9X1, D8L9B5, F4Y593, Q0Q0I7, W5F285, and W5GCA3), and amino acid synthesis (W5AVC9, W5CM54, W5DYX9, W5I8U3, Q45NB6, W5ARG6, and W5ECP3). The DEPs in signal transduction/cell growth could be mainly classified as those involved in phosphorylation (L0GED8), temperature response (P04464 and A4K4Y1), ubiquitin hydrolase (W5B4E9 and W5CWD6) and photosynthesis (P08823, W5B777, and W5ECJ8). The DEPs in defense/stress could be mainly divided into three groups, storage material degradation inhibitors (P16347, P01083, P16159, P17314, P04568, P22701, P42755, Q9ZR70, W5AW62, W5BE38, W5E9F6, W5FHA6, and W5FYW1); response to osmotic, temperature, and oxidative pressure (W5GDM5, C1K737, W5GEI6, Q6W8Q2, W5FJN1, W5FQ55, and W5FDW8); and response to biotic stresses (A1YE31, Q58A30, W5FEK8, Q1XHC6, O64393, Q8GZB0, P82901, and W5FSX7).

### Transcriptional expression analysis as revealed by qRT-PCR

**Fig 7:**
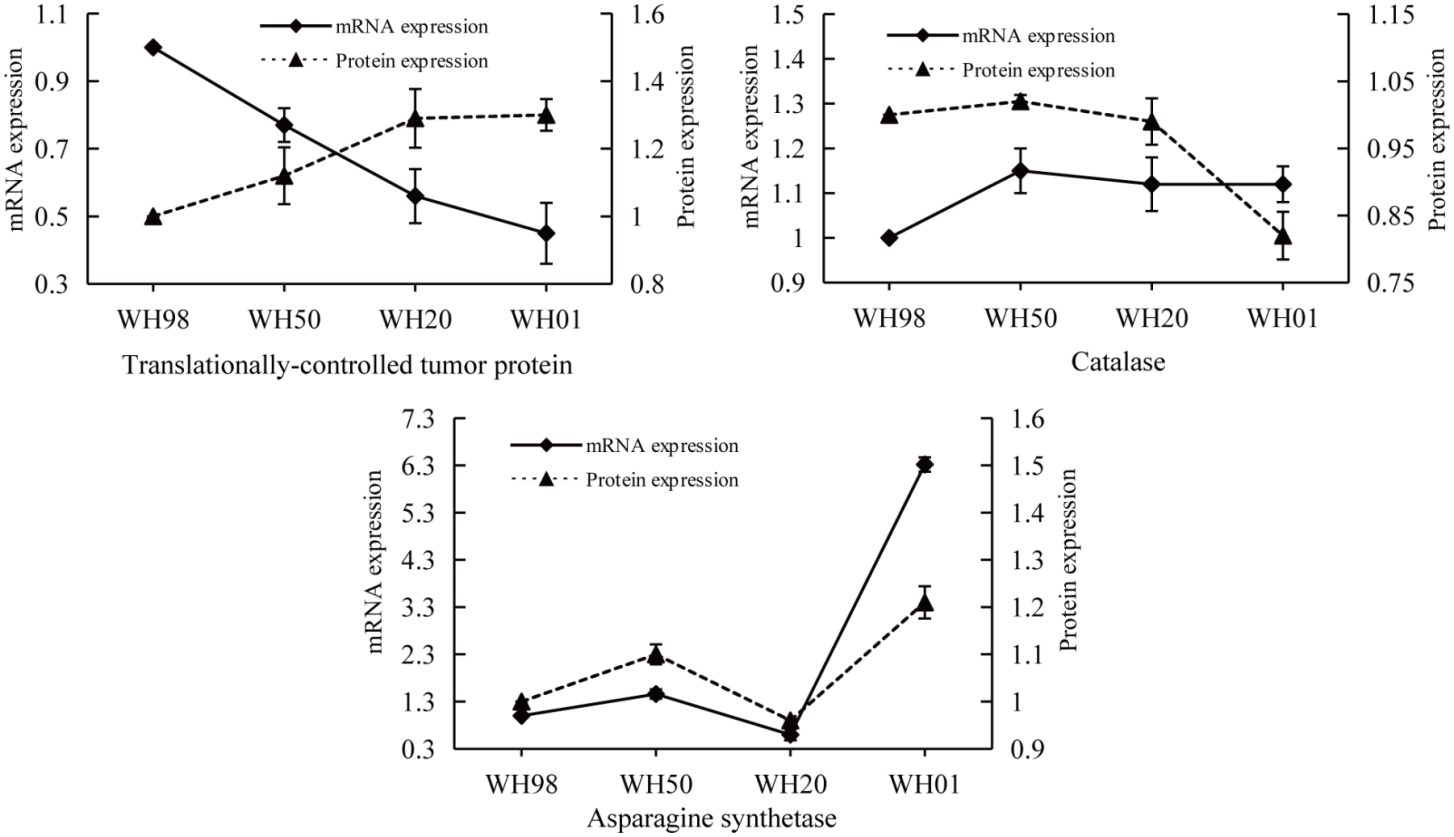
Comparisons of the protein and mRNA expression patterns of three representative DEPs at four artificial ageing stages (WH98, WH50, WH20, and WH01) by iTRAQ and qRT-PCR. Solid lines represent mRNA expression patterns, and dotted lines represent protein expression patterns.

To provide further information on the correspondence between proteins and their mRNA expression patterns, qRT-PCR was performed to investigate the dynamic transcriptional expression patterns of three representative DEPs during artificial ageing. In Fig 7, the gene encoding asparagine synthetase displayed the same expression pattern as its protein. However, the two genes encoding TCTP and catalase showed opposite expression patterns than their proteins. Previous work demonstrated that the expression of TCTP is regulated at both the transcriptional and post-transcriptional levels [39], and such inconsistencies here might be due to post-transcriptional modification. The expression pattern of catalase is similar to that involved in wheat grain development [28], which is regulated by post-transcriptional regulation [40].

## Discussion

The wheat embryo is a significant tissue where seed physiological deterioration and germination begin. In our study, a TMT labeling-based quantitative proteome was employed to detect protein changes during seed artificial ageing and priming. A total of 162 and 531 DEPs were identified during artificial ageing and seed priming, respectively. The DEPs were mainly involved in energy supply, metabolism, and stress response.

### Effects of artificial ageing on wheat seed vigor and proteome

Wheat seed viability was gradually reduced as the Gr decreased from 98% to 1% after 210 d under the artificial ageing conditions used in this study. This effect was similar to the response of maize seeds [4]. In addition, the reduction in Gr was accompanied by structural changes in the embryo. Seed ageing affected the proteome of dry wheat seeds, and such effect is similar to that of maize seeds; this finding confirmed that proteome variation is possible in lowly hydrated seeds [4, 7].

### DEPs involved in seed deterioration

Seed deterioration is a key developmental process during storage. When subjected to artificial ageing, proteins related to energy, storage nutrients, stress, and defense were altered. Several proteins previously reported in other processes were identified for the first time in the aged wheat seeds in this study. This study helps enhance our understanding of biochemical and molecular processes underlying seed deterioration.

#### Metabolism and energy supply

Proteins related to glycolysis, TCA cycle, and PPP significantly contribute to seed vigor [41,42]. In our study, the abundance of 6-phosphofructokinase 1 (W5CP16 and W4ZRX8; the third enzyme and major point of regulation in glycolysis) and glyceraldehyde-3-phosphate dehydrogenase (W5BLN6; the sixth enzyme in glycolysis) decreased. Decreased glyceraldehyde-3-phosphate dehydrogenase levels are also observed in aged Arabidopsis seeds [7]; however, no protein participating in the PPP was detected in our experiment, which was similar to the results of studies on aged Arabidopsis and maize seeds [4,7]. The malic enzyme (malate dehydrogenase, W5A810), responsible for the TCA cycle replenishment pathway, is involved in catalyzing the conversion of pyruvate into malate; this enzyme was down-regulated by seed ageing (Table 1). The changes in malic enzyme was consistent with that in maize and *Arabidopsis* seeds during artificial ageing, indicating that the TCA cycle was disturbed by artificial ageing [4]. In our work, sucrose synthase (W5B5R3 and W5I774) participating in cell respiration and storage deposit [43], and starch branching enzyme 1 (O04074) involved in starch synthesis in plants [44], were also down-regulated. These results signified that the proteins involved in oligosaccharide metabolism were activated in a manner similar to those of maize seeds [4]. No protein involved in the PPP was detected in our study and therefore supported previous works on maize and aged *Arabidopsis* seeds [4,7]. The down-regulated KEGG pathways of energy supply were also enriched (Fig 3D). Kondoh et al. [45] found that enhanced glycolytic activation could inhibit cell senility by protecting against oxidative damage caused by reactive oxygen species, whereas the suppressed glycolytic pathway would result in aged cells. In maize (*Z*. *mays*) seeds, a controlled deterioration treatment inhibited the glycolytic pathway and promoted reactive oxygen species production and accumulation. Then, cell aging or death in seed embryos was accelerated, ultimately leading to seed deterioration and vigor loss [46]. Thus our results indicated that artificially aged wheat seeds triggered responses through the modification of the glycolytic pathway.

#### Storage protein decomposition

Wheat grain proteins accumulate during seed development and decrease with seed ageing. Dell’Aquila previously reported protein change patterns in aged soybean and barley seeds. However, the specific proteins were not identified [47]. In this work, five kinds of storage proteins, particularly glutenin (P10385), avenin-like a1 (Q2A784), gamma-gliadin (P08453), 15 kD grain softness protein (Q43659), and gliadin/avenin-like seed protein (D2KFG9), were down-regulated. These storage proteins are closely related to the quality of wheat flour and thus provide energy for germination [48]. The break-down of these storage proteins might result in an inefficient material supply for the synthesis of new proteins essential for seed germination [16]. A deleterious effect on germination is associated with the protein-synthesizing system [47]. Our results also showed that 60S ribosomal protein L18 (W5A8E0) was up-regulated in aged seeds. Double-stranded RNA-activated protein kinase, a fundamental control step in the regulation of protein synthesis initiation, is negatively regulated by the 60S ribosomal subunit protein L18 in human cells [49]. Determining whether the regulation mechanism in wheat seeds varies will require further study. The KEGG enrichment analysis indicated that up-regulated proteins were associated with the ribosome (Fig 3D) where protein processing occurs. The results might be in accordance with the decreased proteins associated with artificial ageing.

#### Response to biotic and abiotic stimuli

To protect themselves against various stresses, including a biotic or abiotic stimulus, such as oxidative stress, humidity, high temperatures, and pathogen penetration, a number of stress- and defense-related proteins changed during artificial ageing. The decrease in Gr was correlated with the increase of reactive oxygen species, the degradation of storage nutrients, and decreased activity levels of antioxidant enzymes and hydrolases inhibitors, as well as the content of antioxidants, such as L-ascorbic acid and glutathione, after the ageing treatment [5, 9, 50]. In our work, two proteins, catalase (W5HND1) and L-ascorbate peroxidase (W5DYW7), which is related to L-ascorbic acid, were down-regulated. This was consistent with the reduced mitochondrial antioxidant enzyme activities reported in aged soybean seeds [5]. In addition, the KEGG analysis indicated that two kinds of UDP-glucose 6-dehydrogenase (W5FQ55 and W5FDW8) involved in starch, sucrose, and ascorbate metabolism were also down-regulated (S5 and S6 Figs). Previous research indicated that UDP-glucose 6-dehydrogenase could catalyze the biosynthetic oxidation of UDP-glucose into UDP-glucuronic acid [51]. However, its function in oxidative responses has not been reported previously. The alpha-amylase inhibitors may protect stored starch against endogenous amylase activity and protect plants against wounding [23]. In this work, four proteins of alpha-amylase/trypsin inhibitors (P16159, P16851, P17314, and P01083) were down-regulated, indicating that the storage material began to degrade.

Hemoglobin is widely distributed in plants, and plays significant roles in improving the energy metabolism of hypoxic cells and resisting extreme external environmental conditions, such as low temperatures and pathogen attacks [52]. Mirelman et al. [53] reported that wheat germ agglutinin could inhibit fungal growth. In our research, hemoglobin 1 (M5AJV9) and agglutinin isolectin 3 (P10969) were both down-regulated, which signified a decrease in defensive responses against pathogens.

To date, only non-specific lipid-transfer proteins (nsLTPs) were found in plants and their seeds, whereas both LTPs and nsLTPs were discovered in animal tissues [54]. nsLTPs belong to the defense-associated proteins in plants because of their strong activity against plant pathogens [55]. nsLTPs in onion seeds inhibit the growth of *Fusarium solani* [56]; nsLTPs from barley and maize leaves inhibit the growth of *Alternaria brassicola*, *Ascochyta pisi*, *Botrytis cinerea*, *Fusarium culmorum*, and *Verticillium dahliae* [57]; and nsLTPs from *Vigna unguiculata* seeds inhibit the growth of *Fusarium oxysporum* and *F*. *solani* [58]. In our present work, five nsLTPs including P26913, Q2PCC3, W5FSX7, P82901 and W5D2W6, were down regulated, which indicated that the defense capability against pathogen penetration might decrease gradually.

TCTP, a highly conserved protein during evolution, is widely located in eukaryotic cells, including those of plants [59]. TCTP is an important component of the target of rapamycin signaling pathway, the major regulator of cell growth in animals and fungi. Despite its relevance, the molecular functions of plant TCTP homologs remain to be studied. Previous studies showed that the expression of TCTP was enhanced by stress stimuli, including high temperatures and salt stresses in cabbage (*Brassica oleracea L*.) [60], whereas in *Arabidopsis*, TCTP functions as an important growth regulator [59]. Additionally, in animal cells, TCTP functions as a novel heat shock protein with a chaperone-like activity [61]. The multiprotein bridging factor, encoded by *TaMBF1c* in wheat, plays a pivotal role in plant thermo-tolerance and may be useful in improving heat tolerance in other crops [62]. In our study, a TCTP homolog (Q8LRM8) was up-regulated, while the multiprotein bridging factor was down-regulated during the artificial ageing treatment at 45°C. The PPI network analysis indicated that TCTP might interact with other up-regulated proteins during artificial ageing. (S7 Fig and S5 Table). These results suggest that thermo-response factors were triggered when subjected to a high temperature and that the tolerance to high temperature in wheat seeds was balanced by various regulators.

In higher plants, asparagine synthesis is catalyzed by asparagine synthetase, which contributes to the protein synthesis and nitrogen metabolism [63]. Asparagine synthetase is also involved in responding to various factors, such as pathogen infection and oxidative stress [64]. In wheat, deoxynivalenol (DON) is a virulence factor of *Fusarium*, and resistance against DON is considered to be part of *Fusarium* resistance [65]. The evidence for resistance to DON by ribosomal protein L3 (*RPL3*) was claimed by Miller and Ewen [66] and Miller and Arnison [67]. In wheat, six *RPL3* genes, three homologous versions of both paralogs, *RPL3-A* and *RPL3-B*, were expected to be found in *T*. *aestivum* [65]. In our present work, two AS (W5CK94 and W5FDU3) and two *RPL3* (RPL3, A1YE31 and A1YE34) were up-regulated during artificial ageing, indicating that the protection system was triggered in wheat seeds to avoid pathogen penetration.

In plant seeds, storage triacylglycerols (TAG) are present in small discrete intracellular organelles called oil bodies. An oil body has a matrix of TAG, which is surrounded by phospholipids (PL) and alkaline proteins, termed oleosins [68]. Previous research showed that oleosins play important roles in maintaining the structure of the oil body during seed dehydration to facilitate seed germination, promoting the decomposition rate of the oil body during germination, and providing a binding site for lipase during germination [69,70]. In this work, three oleosins (W5AW62, W5FHA6, and W5FYW1) were down-regulated, which might be in accordance with the reduced Gr.

### DEPs participate in seed priming

The priming treatment could accelerate seed germination and improve seedling uniformity. Upon hydro-priming, embryonic cells switch from a quiescent state to a highly active metabolic state. In the present study, many identified proteins might aid in characterizing germination vigor and optimizing germination enhancement treatments.

#### Activated energy supply

The main physiological characteristics during seed priming and subsequent germination are storage degradation, active metabolism, and cell growth [71]. Upon priming with water, storage substances begin to decompose and the central carbon metabolism becomes highly active. Enzymes in glycolysis, the PPP and the TCA cycle are activated during this process. Triosephosphate isomerase (TPI, W5FIN0) is essential for efficient energy production; the enzyme catalyzes the reversible interconversion of dihydroxyacetone phosphate and D-glyceraldehyde 3-phosphate. Meanwhile, pyruvate kinase (W5CVS7), the final enzyme in glycolysis, catalyzes phosphoenolpyruvate to pyruvate, yielding one molecule of ATP [72]. Glucose-6-phosphate-dehydrogenase (W5AU57 and W5B201), the first and also rate-limiting enzyme in PPP, catalyzes the formation of glucose 6-phosphate to form 6-phosphoglucono-δ-lactone, an intramolecular ester that undergoes oxidation. Decarboxylation catalyzed by 6-phosphogluconate dehydrogenase (W5HFZ6), the second enzyme up-regulated in PPP, produces the ketopentose ribulose 5-phosphate (Table 2). The up-regulated malic enzyme (W5A810) in priming catalyzes pyruvate into malate, which is then converted into oxaloacetate, which replenishes the TCA cycle. Our data indicated that the proteins involved in glycolysis, PPP, and TCA were all up-regulated, which was in agreement with those of wheat seed germination [22, 23]. In addition, fructan 1-exohydrolase w2 (*1-FEH w2*, Q84LA1) was up-regulated, being considerably higher during the fructan breakdown phase [73].

Besides the proteins indicated above, fatty acid oxidation is another energy supply pathway. The first step of β-oxidation in the peroxisome is catalyzed by the enzyme acyl-CoA oxidase (*ACOX*, W5DVJ7). This process is also the rate-limiting step in peroxisomal β-oxidation [74]. The up-regulated level of acyl-CoA oxidase indicated that energy was also provided by fatty acid decomposition, which had not been previously reported. These observations demonstrated that energy metabolism was triggered after imbibition to provide energy for subsequent seed germination.

The storage proteins, including globulin-3A (I6QQ39), gamma-gliadin (P04730), and glutenin, and the low molecular weight subunit (P10385) were down-regulated. This finding indicated that they were decomposed to produce energy for seed germination.

The PPI network analysis indicated that the enriched clusters were involved in oxidative phosphorylation and carbon metabolism. Oxidative phosphorylation is the culmination of energy yielding metabolism. All of the oxidative steps in the degradation of carbohydrates, fats, and amino acids converge at this final stage of cellular respiration, in which the energy of oxidation drives the synthesis of ATP [72]. The PPI results indicated that carbohydrates and their oxidative phosphorylation were highly active, and provided energy during seed priming.

#### Physiological metabolism and organizational development

Various physiological metabolic pathways, as well as organizational development, became more active, with fatty acid synthesis, amino acid synthesis, protein translation, and protein folding occurring during this phase. In both eukaryotes and prokaryotes, acetyl-CoA carboxylase is a biotinylated enzyme that catalyzes the first committed step of de novo fatty acid biosynthesis by the carboxylation of acetyl-CoA to malonyl-CoA. In plants, acetyl-CoA carboxylase activity is found in the plastids where primary fatty acid biosynthesis occurs [75]. In plants, monounsaturated fatty acids maintain membrane fluidity and are also sources of storage triacylglycerols in tissues [76]. Acyl-acyl carrier protein (ACP) desaturases are responsible for the conversion of saturated fatty acyl-ACPs into monounsaturated-ACPs. This process is the penultimate step in fatty acid biosynthesis and produces the main source of monounsaturated fatty acids in plant tissues [72,77]. ACP transports the growing fatty acid chain between enzymatic domains of fatty acid synthase during fatty acid biosynthesis [78, 79]. In this work, the accumulation of acetyl-CoA carboxylase (W5DVJ7) involved in fatty acid synthesis was consistent with results of previous research [22]. Acyl-ACP (W5AXD5) and ACP (W5DZ64, W5F3T3, and W5FD02) involved in fatty acid synthesis have not been reported previously during wheat seed priming.

During seed priming and germination, amino acids and proteins accumulate. The aromatic amino acids, as well as other aromatic compounds, which function in plant defense, electron transport, signaling, communication, and wound responses, were synthesized via the shikimate pathway [80, 81]. In our present work, 3-phosphoshikimate 1-carboxyvinyltransferase and chorismate synthase were accumulated. The enzyme 5-enolpyruvylshikimate- 3-phosphate synthase, encoded by *aroA* (3-phosphoshikimate 1-carboxyvinyltransferase), is the sixth enzyme in the shikimate pathway and catalyzes the conversion of phosphoenol-pyruvate and shikimate-3-phosphate into 5-enolpyruvylshikimate 3-phosphate. Meanwhile, chorismate synthase, the seventh enzyme in the shikimate pathway catalyzes the transformation of 5-enolpyruvylshikimate 3-phosphate to the last common precursor, chorismate, in the biosynthesis of numerous aromatic compounds in bacteria, fungi, and plants [72,80,82]. The up-regulated proteins that participated in phenylalanine, tyrosine, and tryptophan biosynthesis are shown in S11 Fig. In addition, enzymes involved in histidine biosynthesis (W5AVC9; histidinol dehydrogenase, last step in histidine biosynthesis) [83], cysteine biosynthesis (W5CM54; cysteine synthase) [84], proline biosynthesis (Q45NB6 and W5ARG6; glutamine synthetase and glutamate dehydrogenase, respectively) [85], and glutamate and asparagine biosynthesis (W5ECP3; *N*-acetyl-gamma-glutamyl-phosphate reductase) [86] were also accumulated. The results suggested that the level of activated amino acid synthesis was similar to that during wheat germination [23].

The active amino acid synthesis pathway provided raw materials for protein synthesis, which might result in the induction of the unfolded protein response (UPR) pathway. In our study, the translation controlling protein eukaryotic translation initiation factor 3 (W5F285 and W5GCA3) [87] was up-regulated. UPR-relevant marker proteins, such as endoplasmic reticulum chaperone BiP (W5GCC9) and protein disulfide isomerase (D8L9B5) were also accumulated during priming. In yeast, the dissociation of Kar2p/BiP from an endoplasmic reticulum sensory molecule, Ire1p, triggers the UPR. The accumulation of BiP suggested that the UPR response might be triggered after priming. Protein disulfide isomerase protein, a multifunctional protein that resides in the endoplasmic reticulum’ lumen, was up-regulated. This enzyme may hold two resulting functions, the formation of molecules with disulfide bonds in wheat, and response to protein stress in the endoplasmic reticulum [88, 89]. Moreover, the highly conserved molecular chaperone 90 kD heat shock protein (F4Y593 and Q0Q0I7), which fulfills a housekeeping function by contributing to protein folding, and a regulatory function by preventing unfolded proteins from aggregating [90], was also up-regulated. Reticulon-like protein, required for endoplasmic reticulum organization [91], was down-regulated in our study, and its function remains to be studied further. The DEPs that participated in protein processing in the endoplasmic reticulum are presented in S8 Fig. In our present work (Table 2), adenylosuccinate synthetase (W5DWA6; involved in purine biosynthesis) and lipoxygenase (W5F9D7; involved in the determination of wheat grain quality) [92] were also up-regulated.

#### Signal transduction/ cell growth

The abundance of 14-3-3 proteins (L0GED8 in Table 2) increased after priming, which was in accordance with a previous study on wheat germination [22]. These proteins are involved in signal transduction chains and facilitate the phosphorylation of target proteins [93]. A calmodulin, which is a type of protein involved in heat shock signal transduction in wheat [94], was found in this study. The α- and β-tubulins are considered important structural elements in cell growth, morphogenesis, and response to cold acclimation [95, 96]. β-Tubulin has been observed in wheat during early germination [93]. In our study, an up-regulated α-tubulin-2A was present, which was in agreement with the report of Gallardo et al. [3]. Two up-regulated ubiquitin carboxyl-terminal hydrolases (W5B4E9 and W5CWD6) were discovered in our study. The ubiquitin carboxyl-terminal hydrolases are a subset of de-ubiquitinating proteases that control biological activity, and regulatory and structural proteins in both plants and animals. In *Arabidopsis*, it contributes to the modification of plant shoot architecture [42]. Another accumulated protein associated with radicle protrusion, S-adenosylmethionine synthase (B0LXM0 and W5G6F1), is synthesized from the pool of stored mRNAs and is involved in the formation of S-adenosyl-methionine, which is essential for plant growth and embryo development [97]. A ribulose-1, 5-bisphosphatecarboxylase/oxygenases large subunit (P08823), two serine hydroxymethyl transferases (W5B777 and W5ECJ8), plasma membrane ATPase (P83970) and fructose-bisphosphate aldolase (W5D043), which are involved in dark reaction/photorespiration/phototropic growth, were found [23, 98–100]. In addition, an up-regulated ferritin (W5ETC7; involved in mineral transport) was also observed [101,94]. These proteins contributed to the onset of the subsequent development of the wheat embryo.

#### Stress response and defense

During seed priming and subsequent germination, various defense proteins stored in mature dry seeds are activated by environmental conditions, including moisture, oxygen, temperature, and oxidative stress [23]. Unlike the down-regulated proteins of L-ascorbic acid, and UDP-glucose 6-dehydrogenase (W5DP16, W5FDW8, and W5FQ55) participating in ascorbate and aldarate metabolisms in aged seeds, they were up-regulated to defend against oxidative stress during priming (S12 Fig and S6 Table). Mak [22] observed marked decreases in the abundances of hydrolytic enzyme inhibitors, including alpha-amylase inhibitor and trypsin inhibitor, in embryos during germination. By contrast, Dong [23] reported that the alpha-amylase inhibitor was abundantly expressed to protect plants against wounding and is beneficial for organ development. In our present work, endogenous alpha-amylase/subtilisin inhibitor (P16347) and alpha-amylase inhibitor 0.28 (P01083) were down-regulated, while alpha-amylase/trypsin inhibitor CM16 (P16159) and CM3 (P17314) were up-regulated. The Em proteins, synthesized at the very early stage in the wheat embryo, may provide a matrix of bound water, which opposes the protein denaturation [102]. In our study, the four Em proteins (P04568, P22701, P42755, and Q9ZR70) were decreased, indicating that protein degradation might be activated. Kibinza [103] indicated that priming induces the synthesis of catalase, which is involved in seed recovery during priming. Up-regulated catalase (W5GEI6) levels were also observed in our study. Previous studies reported that 1-cys peroxiredoxin (Q6W8Q2), which functions as a molecular chaperone under oxidative stress conditions, is down-regulated during seed germination [22]. In our work, 1-cys peroxiredoxin was also down-regulated. Seeds may be exposed to various microorganisms during germination, such as bacterial and fungi. In our present work, several DEPs associated with biotic stresses were identified. These DEPs included ribosomal protein L3-A3 (A1YE31; resistance to DON) [66], P450 (Q58A30; resistance to *Fusarium* head blight) [104], methylenetetrahydrofolate reductase (W5FEK8; infection-related morphogenesis) [105], multidomain cystatin (Q1XHC6; inhibit growth of the snow mold fungus) [106] and Wheatwin-2 (O64393; antifungal activity toward *B*. *cinerea*, *F*. *culmorum* and *Fusarium graminearum*) [107], and nsLTPs (Q8GZB0, P82901, and W5FSX7) [57]. Unlike in artificial ageing, a decreased expression level of multiprotein bridging factor 1 (C1K737), a plant thermo-tolerance protein [62], was observed at normal temperature (25°C) compared with that at higher artificial temperature (45°C). The TCTP was up-regulated during priming, which is consistent with the observation in *Arabidopsis*, in which it functions as an important growth regulator [59]. Chalcone-flavanone isomerase (W5FJN1) in flavonoid biosynthesis has a well-recognized role in the stress response of bread wheat [108–110] and was down-regulated in our research. In addition, proteins, including the up-regulated 3-ketoacyl-CoA synthase (W5GDM5) [111], which is involved in osmotic stress, were also observed in our study. Oleosins, which can promote the decomposition rate of the oil body during germination [69], were down-regulated (W5AW62, W5BE38, W5E9F6, W5FHA6, and W5FYW1), suggesting that oil body decomposition might occur.

### Different physiological states during wheat seed artificial ageing and priming

Seed ageing and seed priming are two distinguishable physiological states of wheat seed. During seed ageing, wheat seeds undergo a metabolic pause and wheat vigor progressively decreases. Upon hydro-priming, embryonic cells switch from a quiescence state to a highly active metabolism state and seed vigor increases rapidly. Therefore, it was important to compare the biochemical behaviors of these two distinct states.

Our present proteome analysis revealed that the enzymes that participated in energy supply displayed opposite changes in the artificial aged and hydro-primed seeds. Enzymes involved in glycolysis (6-phosphofructokinase and glyceraldehyde-3-phosphate dehydrogenase) and the TCA cycle (malic enzyme) were strongly reduced during artificial ageing (Table 1). However, the opposite was observed for proteins involved in glycolysis (triosephosphate isomerase), the PPP pathway (glucose-6-phosphate-dehydrogenase), and the TCA cycle (malic enzyme). Such proteins exhibited strongly increased levels during hydro-priming (Table 2).

Other significant changes observed between ageing and priming involved the stress and response patterns of the seed proteome. Because of the accumulation of reactive oxygen species during seed ageing, antioxidant enzymes, as well as the enzymes participating in antioxidant biosynthesis, including catalase, L-ascorbate peroxidase and UDP-glucose 6-dehydrogenase, were down-regulated. During priming, defense proteins were activated, including UDP-glucose 6-dehydrogenase and catalase. Although our data enumerate the DEPs between ageing and priming, nsLTPs, TCTP, and oleosin remains to be studied further.

Finally, it is remarkable that catabolism was present in the aged seeds, while anabolism was observed in the primed seeds. The abundances of the five kinds of storage proteins including glutenin, avenin-like a1, gamma-gliadin, 15 kD grain softness protein, and gliadin/avenin-like seed protein were decreased. In the primed seeds, several enzymes involved in fatty acid (acetyl-CoA carboxylase, ACP and acyl-ACP), amino acid (shikimate pathway, histidine and proline) and protein synthesis were up-regulated. The up-regulated levels of the endoplasmic reticulum chaperone BiP and protein disulfide isomerase in the UPR pathway confirmed the active status of protein synthesis.

## Conclusions

In summary, wheat embryo during artificial ageing and seed priming was subjected to quantitative proteomic analyses. A total of 162 and 531 DEPs were respectively identified during artificial ageing and seed priming of the ‘Aikang58’ cultivar, and the quantitative expression characterization, functional analysis and protein-protein interactions of these DEPs were investigated.

The DEPs present during artificial ageing participated in various cellular processes, such as protein destination and storage, metabolism and energy supply, and defense/stress, suggesting that the artificial ageing affected these pathways. The reduced ability to protect against ageing may lead to an increase in amylase and protease levels, decomposition of storage substances, impairment of metabolism and energy supply, and ultimately seed deterioration. A considerable number of proteins involved in metabolism and energy supply (malic enzyme, glyceraldehydes-3phosphate dehydrogenase, and 6-phosphofructokinase 1) and defense/stress (catalase, L-ascorbate peroxidase, hemoglobin 1, oleosin, and nsLTPs) were down-regulated. These proteins may play important roles in wheat seed deterioration and might be considered new protein markers for labeling seed ageing. Unlike in seed ageing, most of the DEPs involved in metabolism, energy supply, and signal transduction/ cell growth, were activated. A considerable number of proteins involved in energy supply (glycolysis, the TCA cycle, and the PPP), metabolism (fatty acid oxidation and synthesis, and amino acid synthesis), signal transduction/cell growth (14-3-3 proteins, heat and cold responses), and photorespiration were up-regulated. This pattern is consistent with that of highly active metabolic state. Our results provide comprehensive proteome insights into protein changes that occur during seed deterioration and priming.

The mass spectrometry proteomics data have been deposited to the ProteomeXchange Consortium via the PRIDE [112] partner repository with the dataset identifier PXD004564 and 10.6019/PXD004564.

## Supporting Information

S1 Fig Morphology of seed priming (A) SDS-PAGE analysis of seed embryos proteins during different stages; (B) Mass error distribution of all identified peptides (C); Peptide length distribution (D). (TIF)

S2 Fig Venn diagrams of the identified proteins between three biological repeats (A); Pair-wise pearson’s correlation coefficient of three biology replicates during artificial ageing (B); Protein quantitation reproducibility of three repeat experiment during priming (C). (TIF)

S3 Fig Gene Ontology analysis of DEPs during artificial ageing (A) and priming (B). (TIF)

S4 Fig Functional enrichment analysis of DEPs in artificial ageing. Red bars indicate the up-regulated proteins; green bars indicate down-regulated proteins. GO Ontology enrichment (A, B); KEGG enrichment (C); Domain enrichment (D, E).
(TIF)

S5 Fig Down-regulated proteins (highlighted green boxes) participated in starch and sucrose metabolism during artificial ageing. (TIF)

S6 Fig Down-regulated proteins (highlighted green boxes) participated in ascorbate and aldarate metabolism during artificial ageing. (TIF)

S7 Fig The protein-protein interaction network analysis of up-regulated proteins (A) and down-regulated proteins (B) identified by TMT-labeling during seed artificial ageing. (TIF)

S8 Fig DEPs participated in protein processing in endoplasmic reticulum during seed priming. Red boxes indicate the up-regulated proteins; green boxes indicate down-regulated proteins
(TIF)

S9 Fig Up-regulated proteins (red highlighted boxes) involved in phagosome during priming. (TIF)

S10 Fig Up-regulated proteins (red highlighted boxes) involved in plant-pathogen interaction during priming. (TIF)

S11 Fig Up-regulated proteins (red highlighted boxes) involved in phenylalanine, tyrosine and tryptophan biosynthesis during priming. (TIF)

S12 Fig Up-regulated proteins (red highlighted boxes) involved in ascorbate and aldarate metabolism during priming. (TIF)

S13 Fig Up-regulated proteins (red highlighted boxes) involved in pentose and glucuronate interconversions during priming. (TIF)

S1 Table The identified proteins of three biological replicates and combined data. (XLS)

S2 Table Annotation of the identified proteins. (XLS)

S3 Table Differentially expressed proteins (DEPs) during artificial ageing and priming and primers of DEPs encoding genes for qRT-PCR. (XLS)

S4 Table Functional enrichment analysis (GO, KEGG and Domain enrichment) of DEPs during artificial ageing. (XLS)

S5 Table DEPs involved in protein-protein interaction networks during artificial ageing. (XLS)

S6 Table Functional enrichment analysis (GO, KEGG and Domain enrichment) of DEPs during priming. (XLS)

S7 Table DEPs involved in protein-protein interaction networks during priming. (XLS)
