# Supplementary figures and images for: Quantitative Proteomic Analysis of Wheat Seeds during Artificial Ageing and Priming Using the Isobaric Tandem Mass Tag Labeling
Source: PLoS One. 2016 Sep 15;11(9):e0162851. doi: 10.1371/journal.pone.0162851 (PMC5025167; doi:10.1371/journal.pone.0162851)

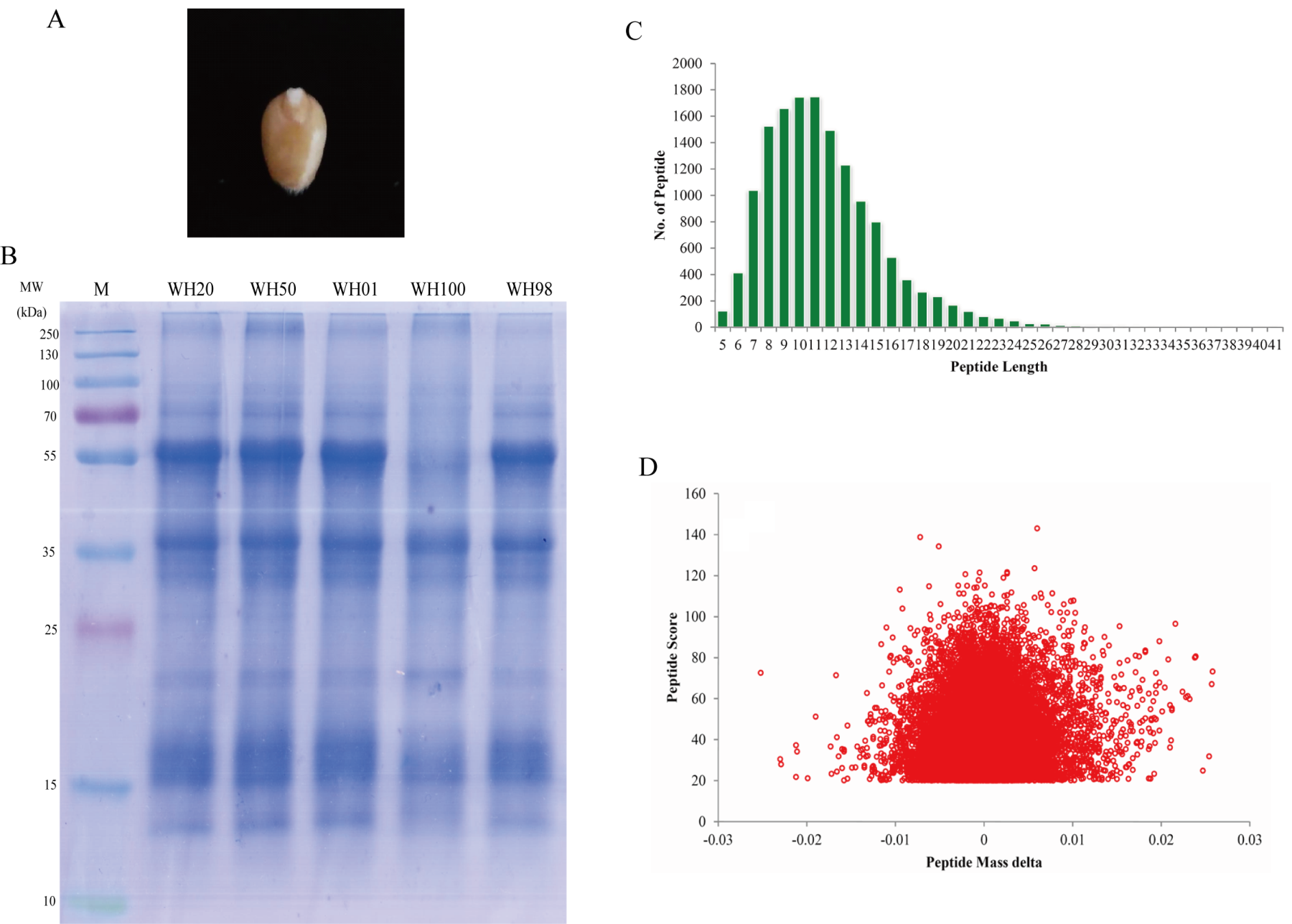

Supplement: S1 Fig — (TIF) [file pone.0162851.s001.tif]

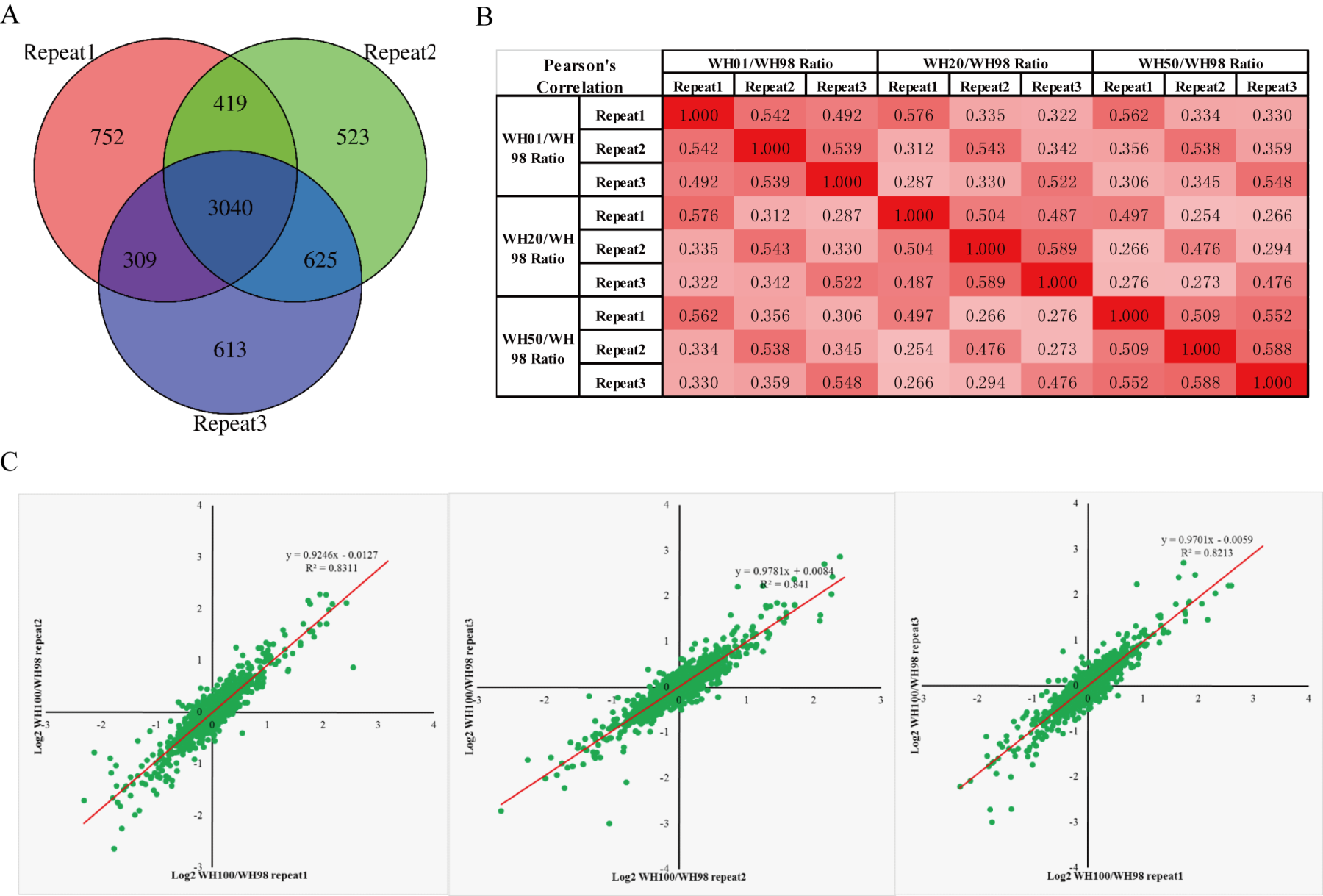

Supplement: S2 Fig — (TIF) [file pone.0162851.s002.tif]

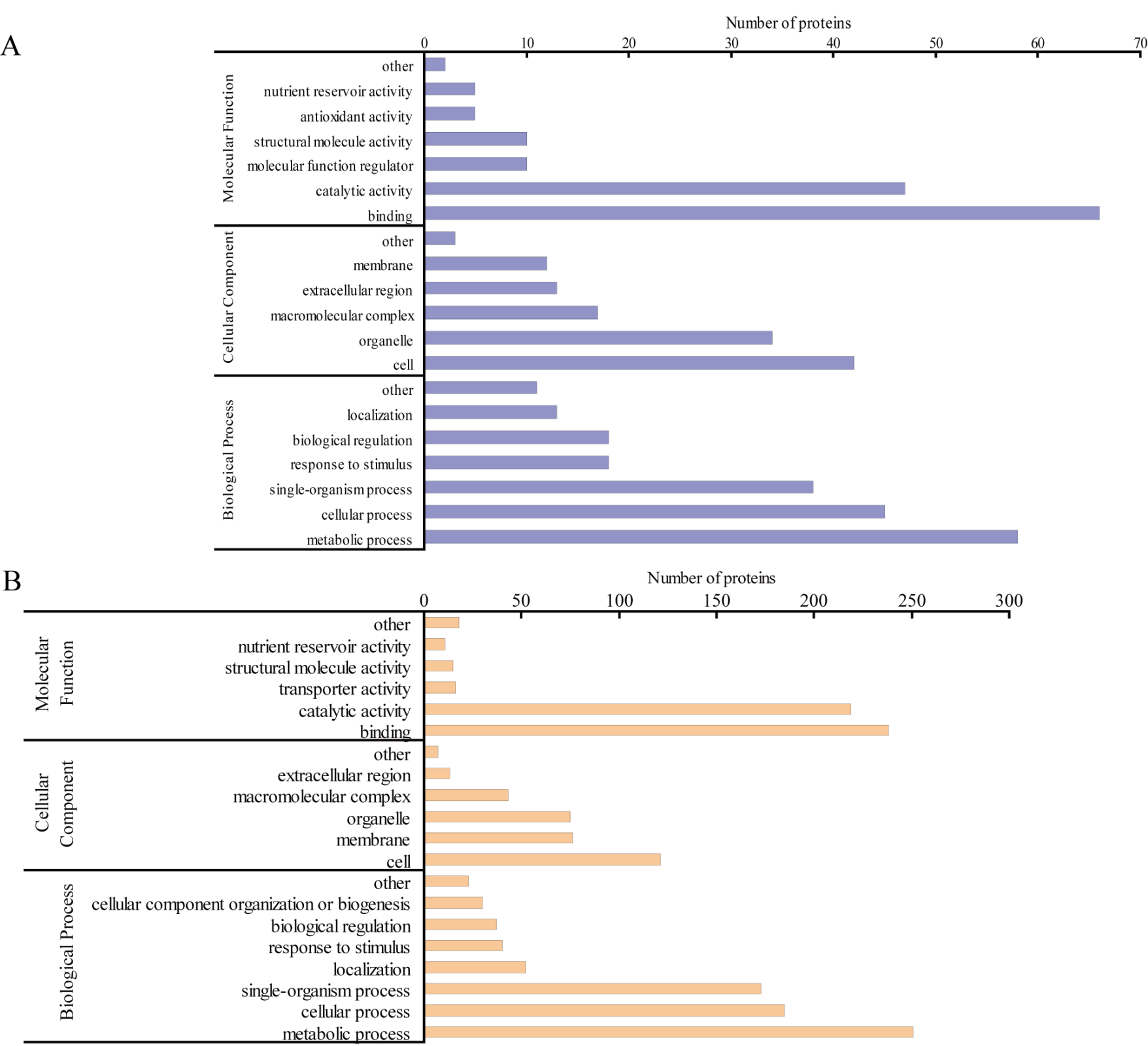

Supplement: S3 Fig — (TIF) [file pone.0162851.s003.tif]

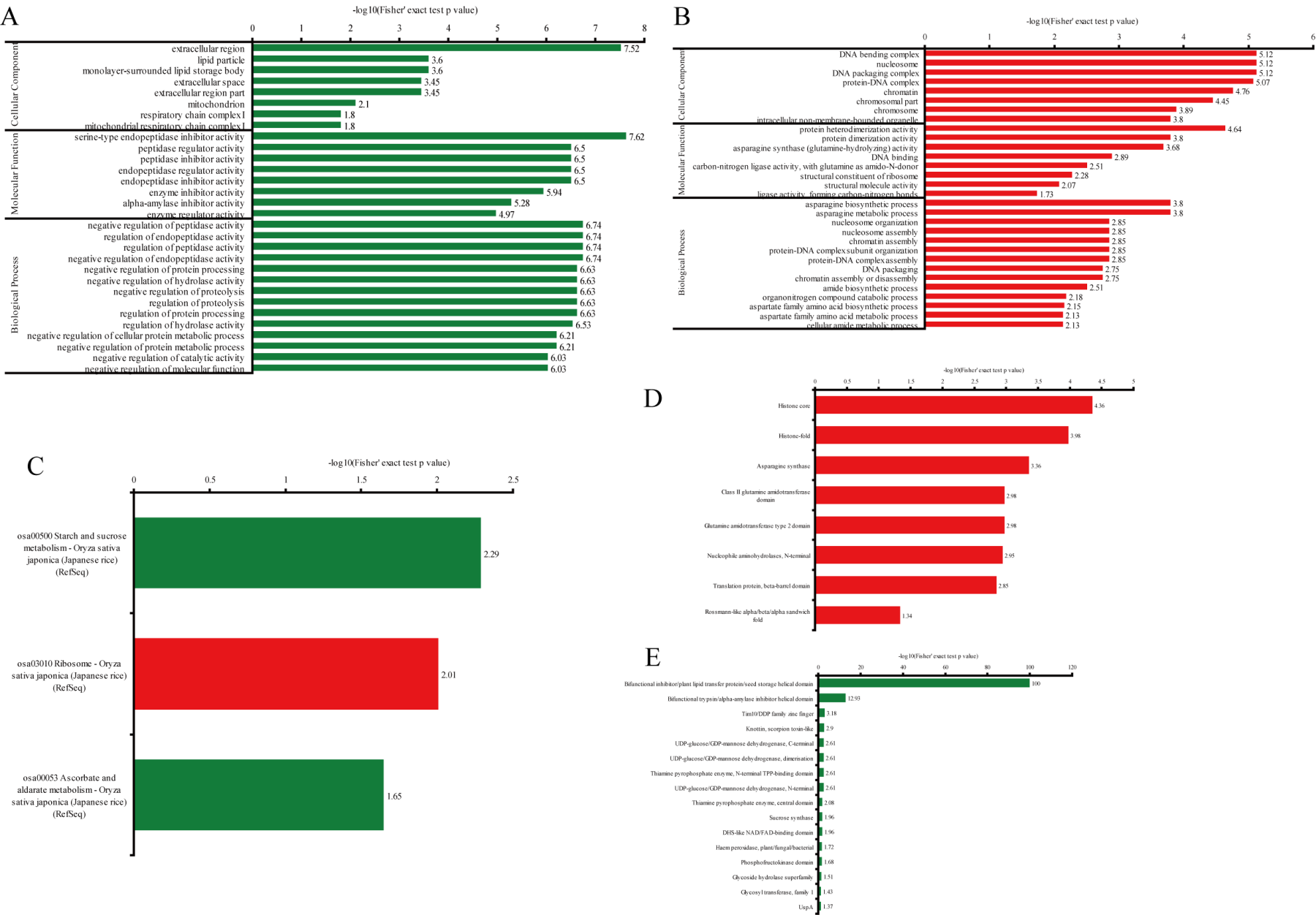

Supplement: S4 Fig — Red bars indicate the up-regulated proteins; green bars indicate down-regulated proteins. GO Ontology enrichment (A, B); KEGG enrichment (C); Domain enrichment (D, E). (TIF) [file pone.0162851.s004.tif]

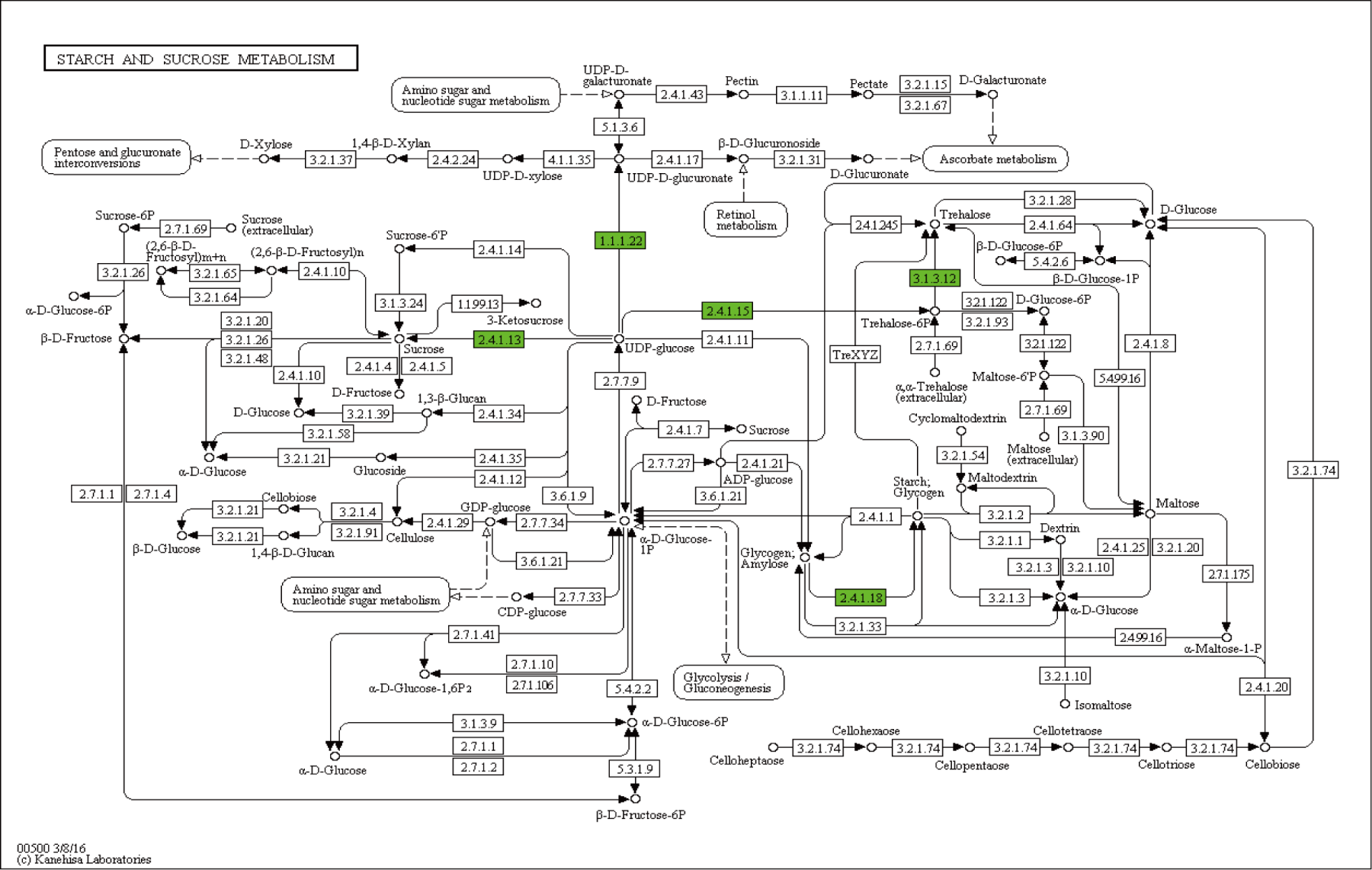

Supplement: S5 Fig — (TIF) [file pone.0162851.s005.tif]

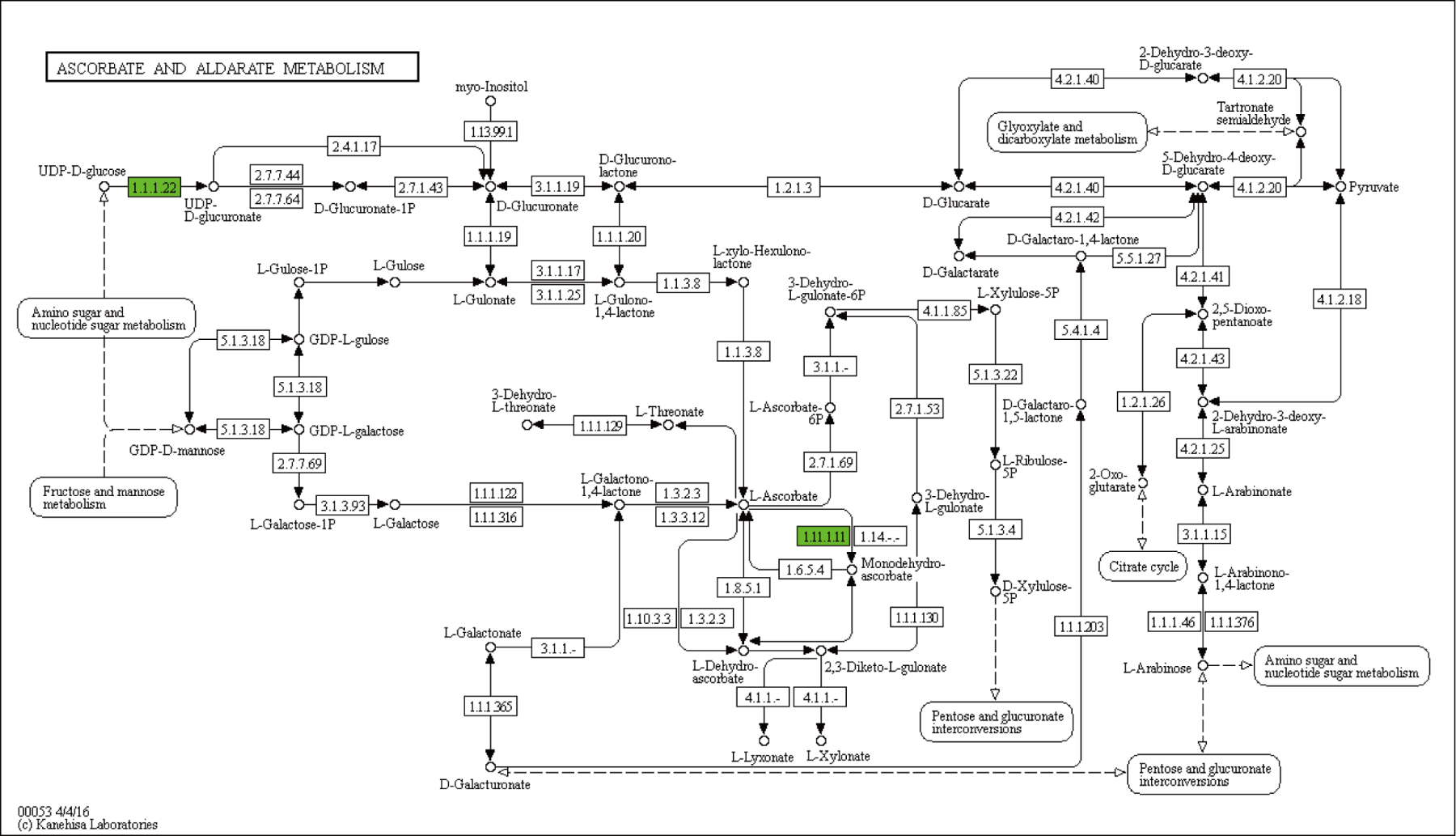

Supplement: S6 Fig — (TIF) [file pone.0162851.s006.tif]

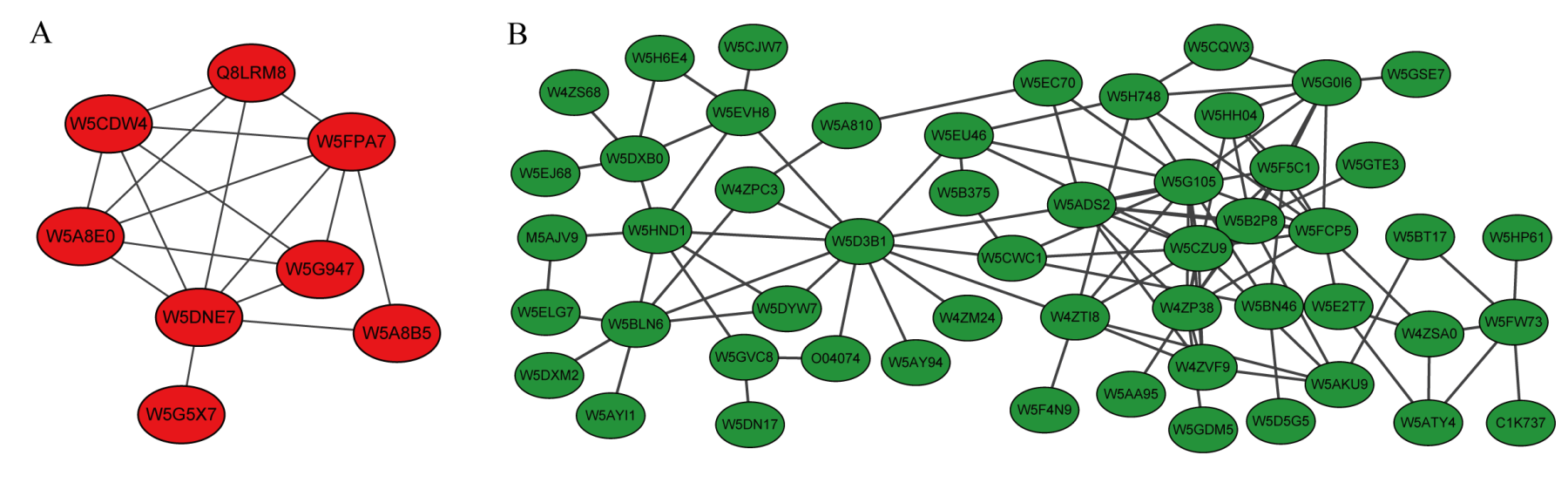

Supplement: S7 Fig — (TIF) [file pone.0162851.s007.tif]

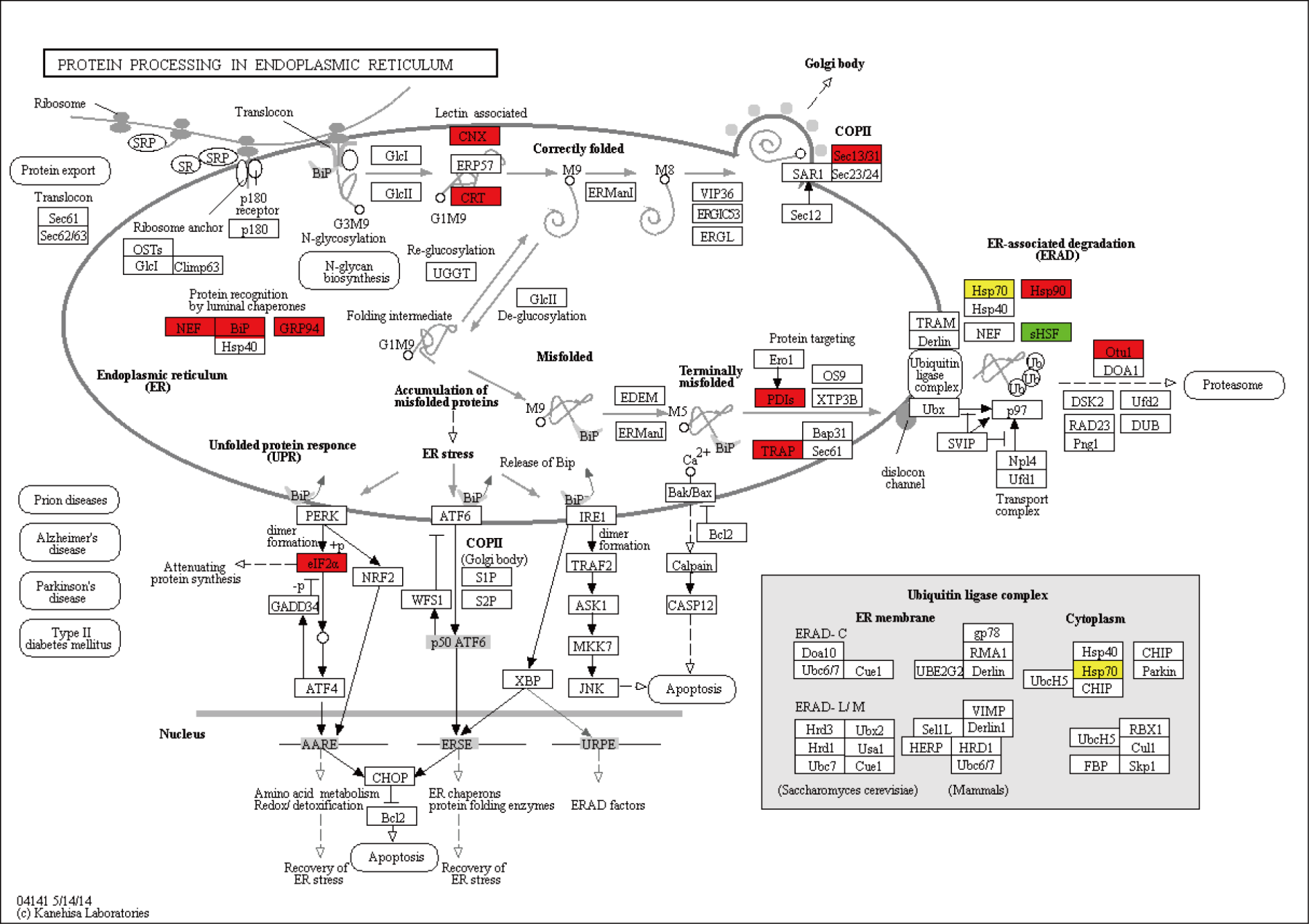

Supplement: S8 Fig — Red boxes indicate the up-regulated proteins; green boxes indicate down-regulated proteins (TIF) [file pone.0162851.s008.tif]

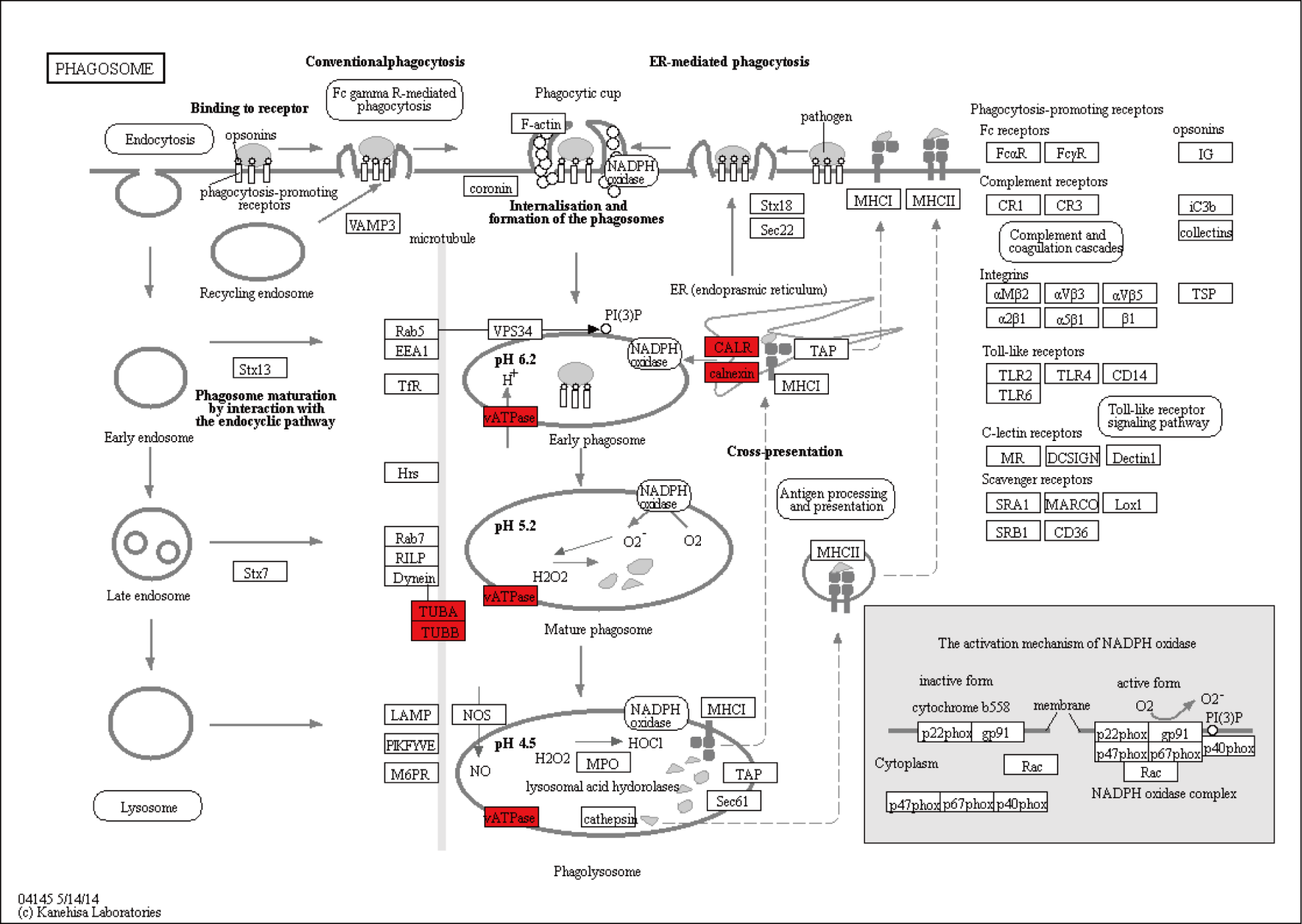

Supplement: S9 Fig — (TIF) [file pone.0162851.s009.tif]

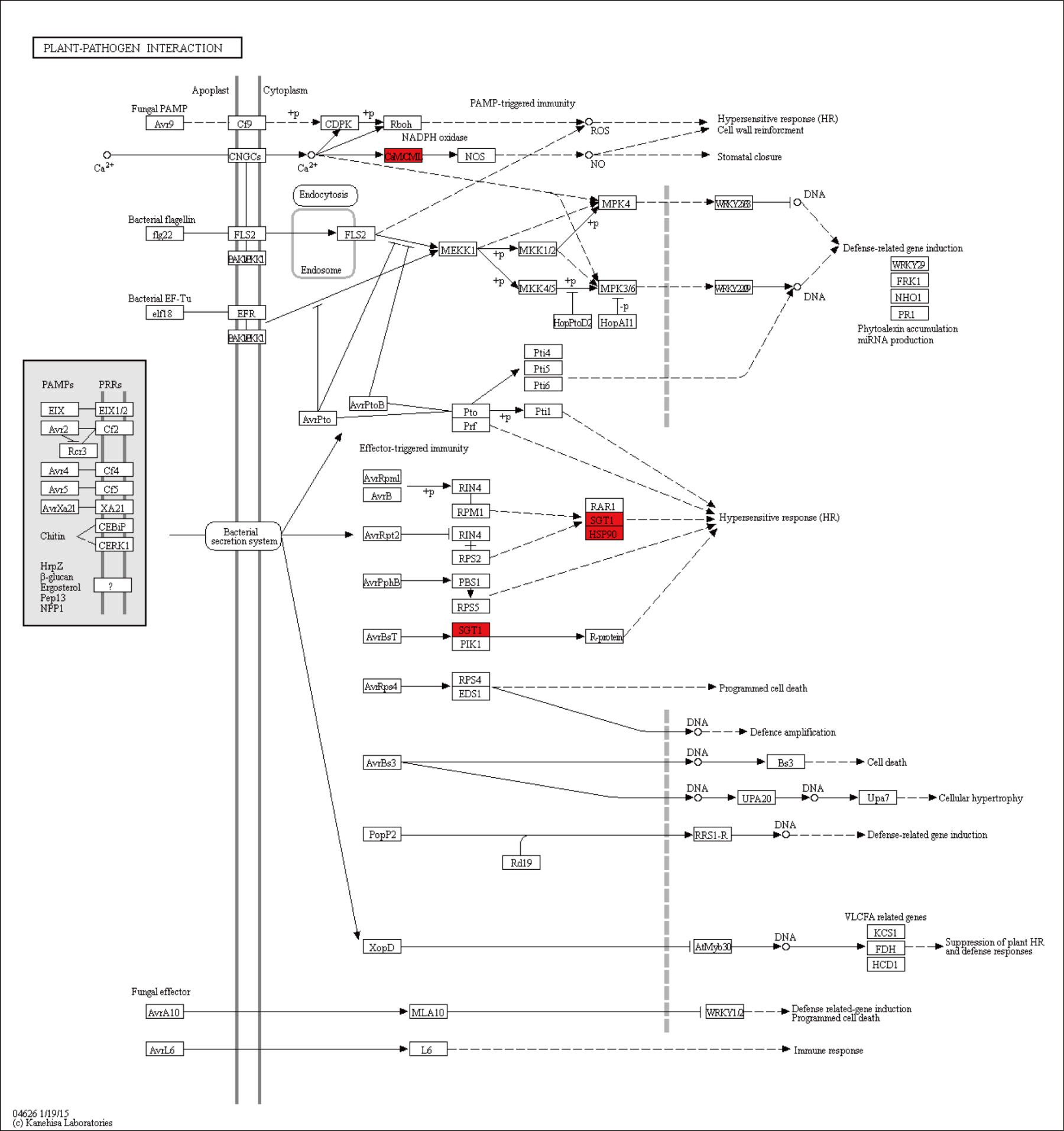

Supplement: S10 Fig — (TIF) [file pone.0162851.s010.tif]

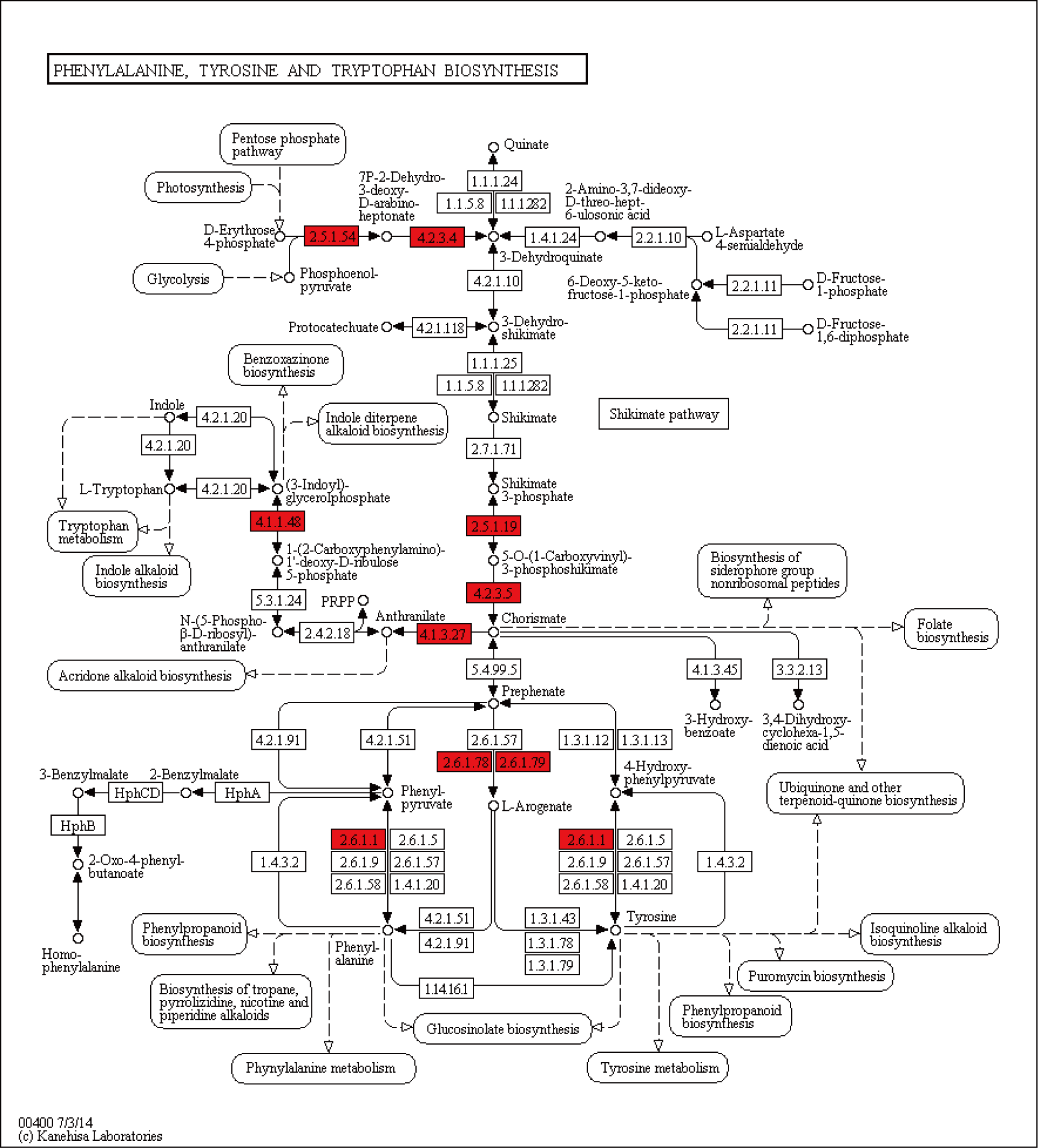

Supplement: S11 Fig — (TIF) [file pone.0162851.s011.tif]

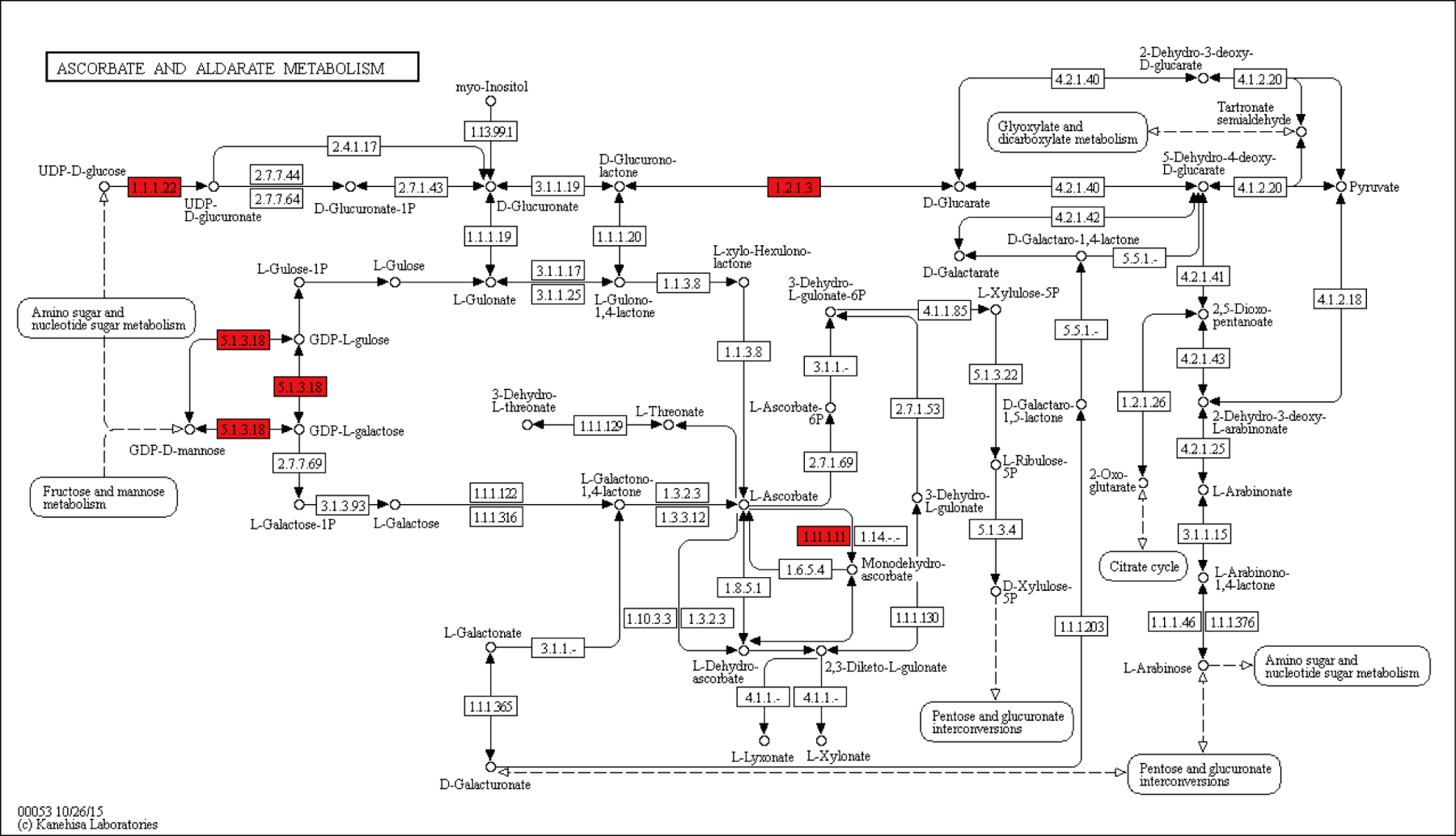

Supplement: S12 Fig — (TIF) [file pone.0162851.s012.tif]

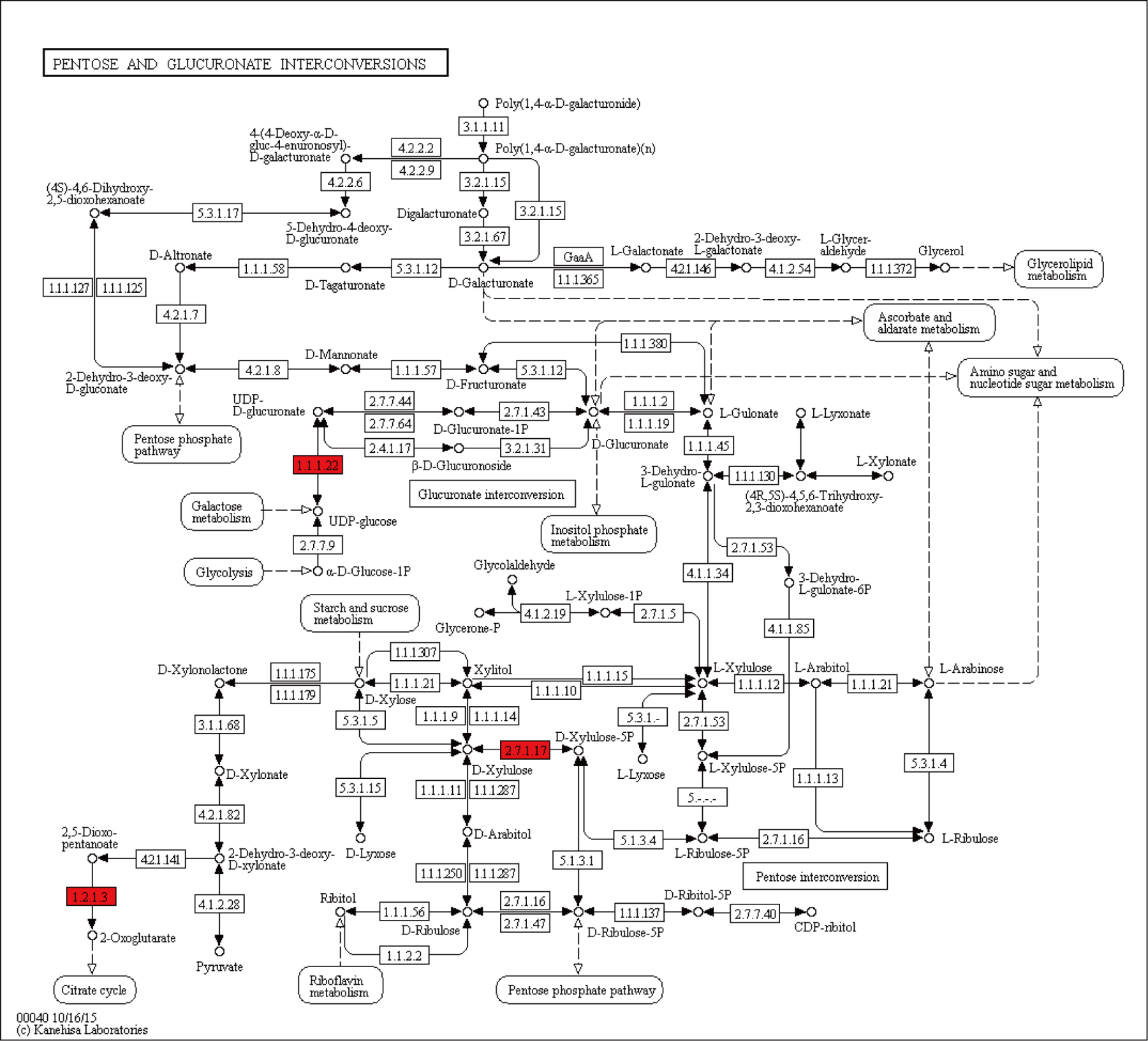

Supplement: S13 Fig — (TIF) [file pone.0162851.s013.tif]
